# Triclosan activates c-Jun/miR-218-1-3p/SLC35C1 signaling to regulate cell viability, migration, invasion and inflammatory response of trophoblast cells in vitro

**DOI:** 10.1186/s12884-022-04791-z

**Published:** 2022-06-06

**Authors:** Weiwei Huo, Ying Wang, Ting Chen, Tianyue Cao, Yue Zhang, Zhouhong Shi, Shunyu Hou

**Affiliations:** 1 Department of Obstetrics and Gynecology, the Affiliated Suzhou Hospital of Nanjing Medical University, Suzhou Municipal Hospital, Gusu School, Nanjing Medical University, Suzhou, Jiangsu China; 2 Suzhou Center for Disease Prevention and Control, Suzhou, Jiangsu China

**Keywords:** Triclosan, miR-218-1-3p, SLC35C1, Inflammatory response, Trophoblast cells

## Abstract

**Background:**

Spontaneous abortion is considered as the commonest complication of pregnancy. Triclosan (TCS) is an antimicrobial agent, which participates in the process of multiple human diseases, including spontaneous abortion. Our study aimed to evaluate the effect of TCS on spontaneous abortion and disclose the possible regulatory mechanism in vitro.

**Results:**

RT-qPCR analyzed that miR-218-1-3p derived from abortion-associated factor slit guidance ligand 2 (SLIT2) was up-regulated in trophoblast cells under TCS treatment. Supported by western blot analysis, functional experiments demonstrated that miR-218-1-3p overexpression impeded the proliferation, migration and invasion while exacerbating the inflammatory response of trophoblast cells. Moreover, mechanism assays revealed that TCS modulated c-Jun production to promote MIR218–1 transcription and enhance miR-218-1-3p expression. Moreover, solute carrier family 35 member C1 (SLC35C1) was validated as a target gene of miR-218-1-3p, and miR-218-1-3p was sustained to negatively modulate SLC35C1 expression in trophoblast cells. Rescue assays validated the role of TCS/miR-218-1-3p/SLC35C1 axis in regulating the viability, migration, invasion and inflammatory response of trophoblast cells.

**Conclusions:**

TCS regulated miR-218-1-3p/SLC35C1 axis to modulate the proliferation, migration, invasion and inflammatory response of trophoblast cells in vitro, which might provide novel insights for spontaneous abortion prevention.

**Graphical Abstract:**

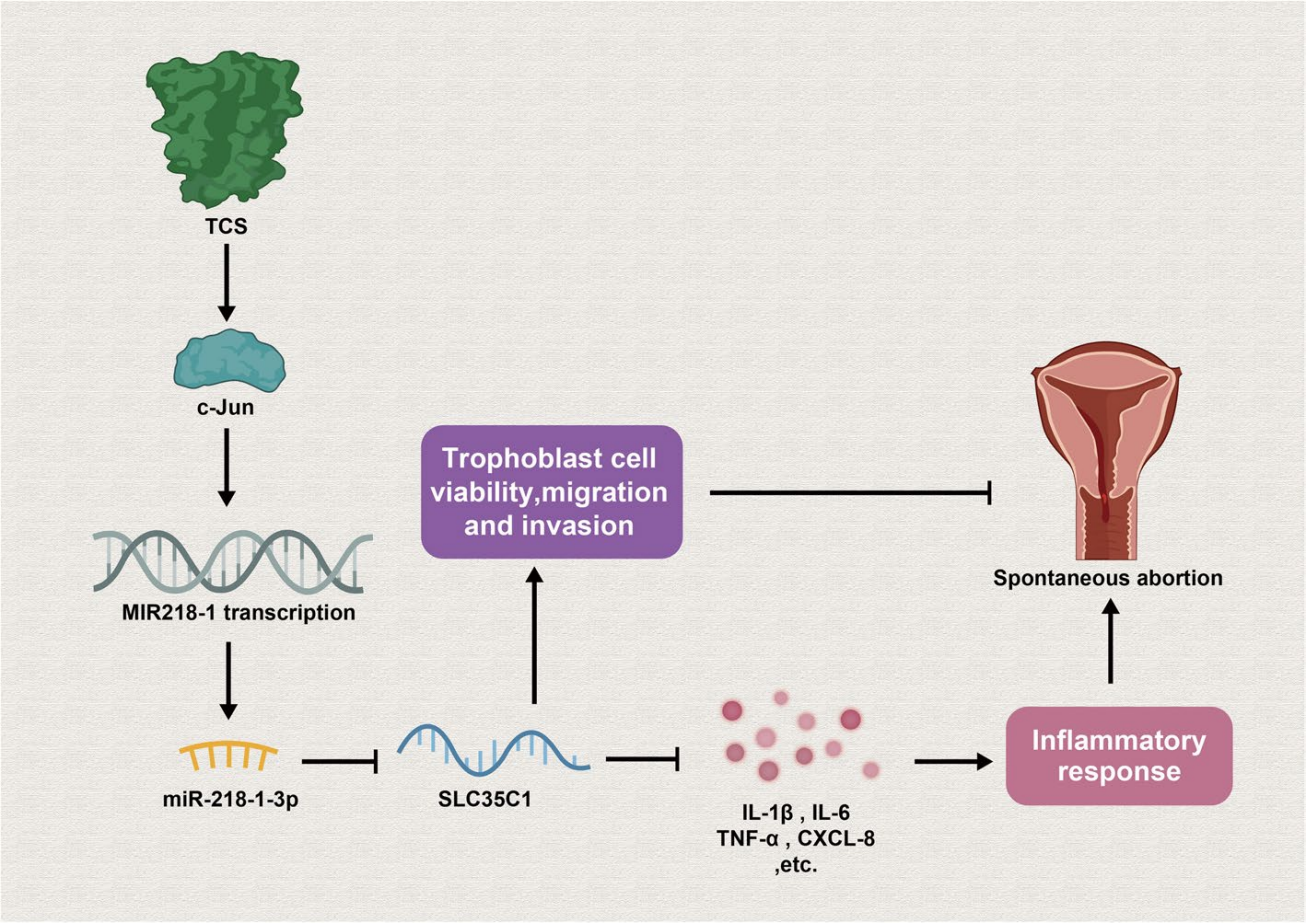

**Supplementary Information:**

The online version contains supplementary material available at 10.1186/s12884-022-04791-z.

## Background

Spontaneous abortion is defined as the loss of a pregnancy without outside intervention before 20 weeks’ gestation [1]. It is the commonest complication of pregnancy with an incidence estimated at 10–15% [2, 3]. For clinical purposes, spontaneous abortion could be divided into several recognized types including threatened abortion, inevitable abortion, incomplete abortion, complete abortion, missed abortion and septic abortion [4]. There are several risk factors prior to pregnancy associated with spontaneous abortion, which are complex and modifiable, including menarche age, family genetic diseases and maternal congenital defects [5]. Moreover, more women are vulnerable to mental health problems after the occurrence of spontaneous abortion, especially for immigrants, childless women and women in a low socioeconomic status [6]. Meanwhile, we have to recognize that dysfunction of trophoblast cells is responsible for the abortion [7], and inflammatory response is involved in abortion [8].

Triclosan (TCS) is an antimicrobial which is frequently applied in toothpaste, mouthwash, hand sanitizer, and surgical soaps [9]. TCS has been sustained as an essential effector in human diseases in accumulating studies. For instance, TCS have been proposed to induce human vascular endothelial cell injury via modulation on PI3K/Akt/mTOR [10]. TCS exacerbates the development of steatohepatitis and fibrosis, coupled with an increment in levels of hepatic lipid droplets and oxidative stress [11]. Liu et al. have demonstrated that enough concentrations of TCS are capable of inhibiting the viability and growth of breast cancer cells (MCF-7 and SKBr-3) in culture [12]. Meanwhile, Wang et al. have revealed that patients who underwent spontaneous abortion had much higher urinary TCS (over 10 folds) than normal pregnancy, and TCS exposure in mice may lead to spontaneous abortion in mid-gestation [13]. However, the way in which TCS modulated the process of spontaneous abortion needs to be investigated.

MicroRNAs (miRNAs) are short non-coding RNAs which play an important role in regulating gene expression and the implication of aberrantly expressed miRNAs in tumorigenesis and progression of malignancies has been unveiled in previous papers [14, 15]. For instance, miR-93 influences cell proliferation and apoptosis in recurrent spontaneous abortion via targeting BCL2L2 [16] and miR-181b-5p has been suggested to exert suppressive impacts on trophoblast cell migration and invasion via directly targeting S1PR1 [17]. Moreover, the association between TCS and miRNAs in human diseases has also been mentioned in previous research. For instance, Ha et al. have presented that miR-6321 induced by TCS is implicated in testicular steroidogenesis, and specifically, miR-6321/Map 3 k1-mediated JNK/c-Jun/Nur77 cascade contributes to TCS-induced inhibition on testicular steroidogenesis [18]. However, miRNAs influenced by TCS in trophoblast cells require further exploration.

In this paper, the main aim was to uncover the role of TCS in spontaneous abortion in vitro and explore the underlying downstream regulatory mechanism of TCS.

## Methods

### Cell culture

Human trophoblast cells (JEG3 and HTR-8) and human embryonic kidney 293 T (HEK-293 T) were selected for this study. Both JEG3 and HTR-8 showed epithelial-like phenotypes under a light microscope (Supplementary file 1). JEG3 cell line was obtained from Huatuo Biotechnology Co., Ltd. (Shenzhen, China) while HTR-8 and HEK-293 T cells were purchased from American Type Culture Collection (ATCC; Manassas, VA, USA). JEG3 cells were incubated in EMEM Medium containing 10% FBS (Gibco). HTR-8 cells were maintained in RPMI-1640 Medium containing 5% FBS while HEK-293 T cells were cultured in Dulbecco’s Modified Eagle’s Medium (DMEM) containing 10% FBS. All mediums were kept under the condition of 5% CO_2_ at 37 °C. Cell cultures were regularly checked by polymerase chain reaction (PCR) for mycoplasma contamination, as previously described [19]. Additionally, the authentication of JEG3 and HTR-8 cells, as well as cross-contamination, was checked by STR profiling, and the corresponding STR reports were provided in Supplementary files 2 and 3.

### Plasmid transfection

Genechem (Shanghai, China) synthesized the short hairpin RNAs (shRNAs) targeting JUN or SLC35C1, as well as sh-NC. In addition, pcDNA3.1-JUN and the empty vector were also obtained from Genechem while miR-218-1-3p mimics/inhibitor and negative control were procured from RiboBio (Shanghai, China). Transfection of the above-mentioned plasmids was completed with Lipofectamine 3000 (Invitrogen). The transfection efficiency was ranged from 75 to 85%.

### Quantitative real-time PCR (RT-qPCR) analysis

Firstly, total RNA was obtained with TRIzol Reagent (Invitrogen, Carlsbad, CA, USA). Then M-MLV reverse transcriptase (Promega, Madison, USA) was employed to convert total RNA into cDNA. Through using SYBR Green Real-Time PCR Kit (Takara), RT-qPCR was performed. All target genes were measured with 2^−ΔΔCt^ method and GAPDH or U6 was considered as the internal control. The Ct values obtained from real-time PCR analyses for indicated genes under different conditions were presented in Supplementary file 4.

### Cell counting kit-8 (CCK-8) assay

At first, cells were incubated into 96-well plates (5 × 10^3^ cells/well). After the addition of CCK-8 solution to each well, the optical density at 450 nm at indicated time points (0, 24, 48, 72 h) was measured with a spectrophotometer (Thermo Fisher Scientific, Waltham, MA, USA) after additional 2-h incubation to reflect changes in cell viability.

### Transwell assay

In detail, the upper chambers of Transwell inserts (Corning Incorporated, Corning, NY) with serum-free medium were used to accommodate trophoblast cells (5 × 10^4^ cells). Transwell inserts covered by Matrigel was used for invasion assay. Then, the basal chamber was supplemented with culture medium containing 10% FBS. After being dyed by crystal violet, cells were counted 24 h later.

### Wound healing assay

JEG3 and HTR-8 cells were plated in six-well plates with 5 × 10^5^ cells/well and grown in complete culture media. After reaching 80% confluence, cells were starved for 24 h. Then, the surface of each well was lightly wounded using a sterile micropipette tip, and the cells were treated with complete culture media again. An inverted optical microscope (× 200) (Nikon, Japan) was used to observe and monitor the wound width at 0, 6, 12, 18, 24 h. The closure rate was defined as per the formula: closure rate = (wound width at 0 h – wound width at indicated time)/wound width at 0 h.

### Chromatin immunoprecipitation (ChIP)

EZ ChIP™ Chromatin Immunoprecipitation Kit (Millipore, Burlington, MA, USA) was used for ChIP assay. Briefly, the crosslinked chromatin DNA was sonicated to obtain chromatin fragments which were precipitated with anti-c-Jun (ab32137, 1/50 dilution, Abcam, Cambridge, MA, USA) antibody and immunoglobulin G (IgG) antibody (#3900, 1/50 dilution, Cell signaling Technology, Boston, MA, USA). The immunoprecipitated DNA was subjected to PCR analysis.

### Luciferase reporter assay

The MIR218–1 promoter region containing the binding sites (wild type or mutant type) of JUN was constructed into the pGL3 vector (Promega, Madison, WI) and co-transfected along with pcDNA3.1-JUN or the empty vector into JEG3 and HTR-8 cells. Similarly, SLC35C1 3’UTR with wild type or mutant type miR-218-1-3p binding sites were sub-cloned into pmirGLO vectors and then the constructs were co-transfected with NC mimics or miR-218-1-3p mimics into HEK-293 T cells. After 48 h, the Dual-Luciferase Reporter Gene Assay Kit (Yeasen, Shanghai, China) was applied to measure luciferase activity.

### Western blot analysis

First of all, proteins in cells were extracted by RIPA lysis buffer (Sigma-Aldrich, St. Louis, MO, USA) and then concentrated via bicinchoninic acid (BCA) kit (Thermo Fisher Scientific). Next, total of 20 μg proteins in RIPA buffer were added into each well of the gel, and were then separated by 12% SDS-PAGE. After that, proteins were transferred onto PVDF membranes (Millipore, Billerica, MA, USA), and the membranes were then blocking with 5% non-fat milk. Afterwards, membranes were cropped as needed and then incubated by primary antibodies at 4 °C overnight, followed by PBS washing for 3 times and 1-h incubation with secondary antibody at dark room. The antibodies against IL-6 (ab233706, 1/1000 dilution, Abcam), IL-1β (ab216995, 1/1000 dilution, Abcam), TNF-α (ab66579, 1/1000 dilution, Abcam), CXCL-8 (ab154390, 1/2000 dilution, Abcam), c-Jun (ab32137, 1/2000 dilution, Abcam), SLC35C1 (ab60336, 1.25 μg/mL, Abcam) and GAPDH were all bought from Abcam. Lipopolysaccharide (LPS)-treated HUVEC whole cell lysate (LPS-HUVEC-WCL), LPS-treated THP-1 whole cell lysate (LPS-THP-1-WCL), HeLa whole cell lysate (HeLa-WCL; ab150035) and human Jurkat cell lysate (Jurkat-CL) were used as positive controls for antibodies against IL-6, IL-1β/TNF-α, CXCL-8/c-JUN and SLC35C1, respectively. Protein quantification was conducted by Immobilon Western Chemiluminescent HRP Substrate (Merck Millipore, Billerican MA, USA). The original blots with clear edges and marker band sizes were provided in Supplementary file 5.

### Statistical analysis

SPSS version 16.0 (IBM, Chicago, IL) was used to analyze data. The data are displayed as the mean ± SD. All experiments were conducted in triplicate. Unpaired two-tailed Student’s t-test or ANOVA was used to analyze the differences between two or more groups. *P* value less than 0.05 was thought to be statistically significant.

## Results

### TCS impedes the proliferation, migration and invasion while stimulates the inflammatory response of trophoblast cells in vitro

To evaluate the influence of TCS on trophoblast cells, we treated trophoblast cells with different concentrations of TCS and detected cell viability. The results of CCK-8 assay demonstrated that the viability of trophoblast cells decreased more with the increasing concentrations of TCS, and when treated with 10 μM TCS, the viability of trophoblast cells showed obvious decrease (Supplementary Fig. 1A, *P <* 0.01). Based on this finding, 10 μM TCS was selected for subsequent functional assays. Through wound healing and transwell assays, the migratory and invasive capacities of trophoblast cells were discovered to be attenuated under the treatment of TCS (Supplementary Fig. 1B-C, *P <* 0.01). Additionally, RT-qPCR and western blot results indicated that the expression levels of proinflammatory factors (IL-6, IL-1β, TNF-α and CXCL-8) were all enhanced after TCS treatment (Supplementary Fig. 1D-H, *P <* 0.01). In conclusion, TCS hinders trophoblast cell proliferation, migration and invasion while inducing inflammatory response.

### MiR-218-1-3p up-regulation induced by TCS influences the proliferation, migration, invasion and inflammatory response of trophoblast cells in vitro

**Fig. 1:**
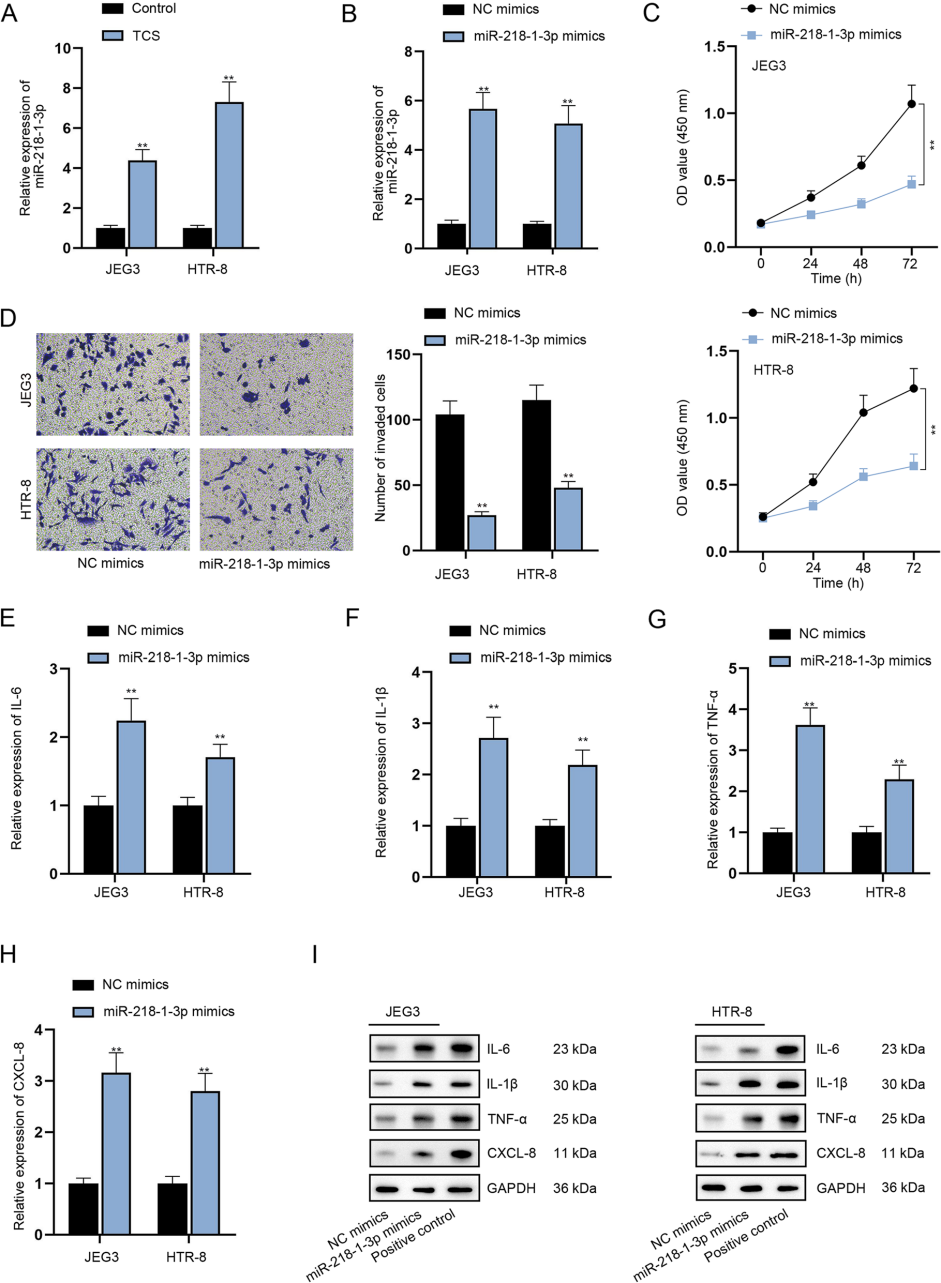
MiR-218-1-3p induced by TCS influences the proliferation, invasion and inflammatory response of trophoblast cells in vitro. **A** RT-qPCR tested miR-218-1-3p expression after TCS treatment. **B** RT-qPCR examined the efficiency of miR-218-1-3p mimics. **C-D** CCK-8 and transwell assays evaluated cell proliferation and invasion. **E-I** RT-qPCR and western blot analyzed the expression of IL-6, IL-1β, TNF-α and CXCL-8 in cells with or without miR-218-1-3p overexpression. The samples in the last group of western blot experiment were indicated positive controls for corresponding antibodies. ^**^P < 0.01

SLIT2 has been reported to be associated with early pregnancy and miscarriage [20]. Moreover, miR-218-1-3p is embedded in introns of SLIT2 [21]. Interestingly, through RT-qPCR analysis, we discovered that miR-218-1-3p expression was enhanced after the addition of TCS (Fig. 1A, *P <* 0.01). Hence, we speculated that TCS might influence miR-218-1-3p expression to participate in the process of spontaneous abortion. To verify the hypothesis, gain-of-function assays were conducted to examine the influences of miR-218-1-3p on trophoblast cells. To overexpress miR-218-1-3p, JEG3 and HTR-8 cells were transfected with miR-218-1-3p mimics (Fig. 1B, *P <* 0.01). The experimental results of CCK-8 assay revealed that up-regulation of miR-218-1-3p inhibited the proliferation of trophoblast cells (Fig. 1C, *P <* 0.01). Wound healing assay results revealed that the trophoblast cell migratory capability was weakened by miR-218-1-3p augment (Supplementary Fig. 1I, *P <* 0.01). Consistently, miR-218-1-3p overexpression hindered the invasion of JEG3 and HTR-8 cells (Fig. 1D, *P <* 0.01). Further, RT-qPCR and western blot analysis suggested that when miR-218-1-3p was up-regulated, the mRNA and protein levels of IL-6, IL-1β, TNF-α and CXCL-8 were all increased (Fig. 1E-I, *P <* 0.01), which implied that miR-218-1-3p overexpression contributed to the inflammatory response of trophoblast cells. To conclude, miR-218-1-3p inhibits the proliferation, migration and invasion of trophoblast cells while promoting the inflammatory response in vitro.

### TCS-induced c-Jun transcriptionally up-regulates miR-218-1-3p

**Fig. 2:**
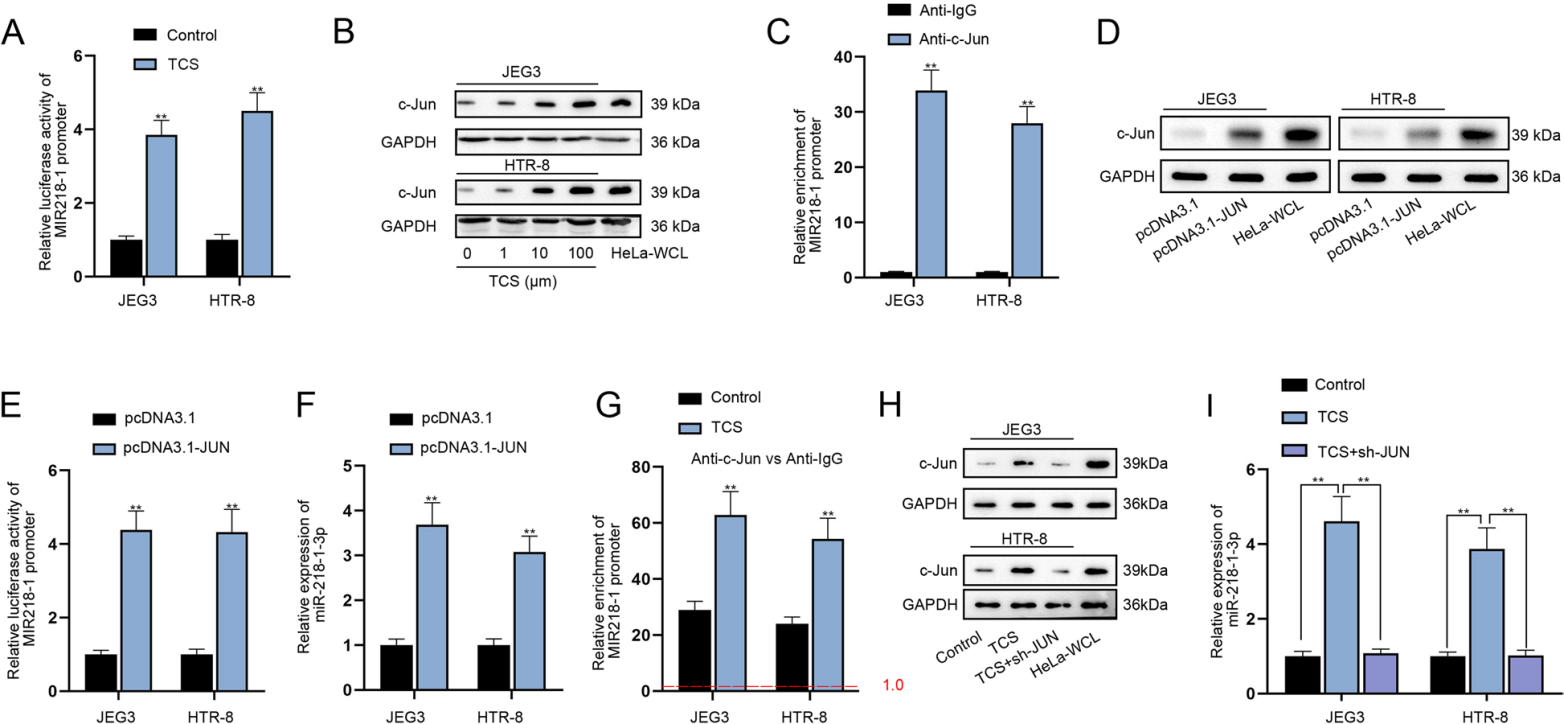
TCS induces c-Jun to up-regulate miR-218-1-3p at transcriptional level. **A** Luciferase reporter assay examined the luciferase activity of MIR218–1 promoter after TCS treatment. **B** The protein level of c-Jun was analyzed by western blot in trophoblasts under different concentrations of TCS. **C** ChIP assay verified the interaction between c-Jun and MIR218–1 promoter. **D** Western blot examined the efficiency of pcDNA3.1-JUN. **E** The luciferase activity of MIR218–1 promoter was tested after JUN was overexpressed. **F** MiR-218-1-3p expression was examined after transfection of pcDNA3.1-JUN. **G** ChIP assay detected the abundance of MIR218–1 promoter in c-Jun antibody under the treatment of TCS. **H** Western blot analyzed the protein levels of c-Jun in the Control group, TCS group and TCS + sh-JUN group. **I** RT-qPCR analyzed the expression of miR-218-1-3p in indicated groups. ^**^P < 0.01

With the application of UCSC database (http://genome.ucsc.edu/), we noticed that c-Jun might interact with the promoter of MIR218–1. As reported, c-Jun production may be influenced by TCS [22]. Hence, we speculated that TCS might regulate miR-218-1-3p via affecting the production of c-Jun. To verify this conjecture, luciferase reporter assay was implemented and the results manifested that TCS treatment increased the luciferase activity of MIR218–1 promoter (Fig. 2A, *P <* 0.01), indicating that TCS activated the transcription of MIR218–1. Moreover, the addition of 10 μM TCS obviously elevated the protein level of c-Jun and the influence became more apparent with the increasing concentration of TCS (Fig. 2B). Moreover, ChIP assay validated that MIR218–1 promoter was abundantly precipitated in c-Jun antibody (Fig. 2C, *P <* 0.01), implying that c-Jun interacted with MIR218–1 promoter. To enhance c-Jun expression, pcDNA3.1-JUN plasmid was transfected into JEG3 and HTR-8 cells (Fig. 2D). Luciferase reporter assay confirmed that pcDNA3.1-JUN transfection led to increased luciferase activity in cells with co-transfection of pGL3 reporters carrying MIR218–1 promoter (Fig. 2E, *P <* 0.01), implying that c-Jun might facilitate the transcription of MIR218–1. Furthermore, an augment in miR-218-1-3p expression was discovered as a result of pcDNA3.1-JUN transfection (Fig. 2F, *P <* 0.01). Moreover, we discovered that through ChIP assay that under the treatment of TCS, the binding of c-Jun to MIR218–1 promoter was strengthened (Fig. 2G, *P <* 0.01). Additionally, western blot analysis indicated that TCS enhanced the protein level of c-Jun while this effect was completely restored by JUN silencing (Fig. 2H). Also, the increase in miR-218-1-3p expression induced by TCS treatment was completely reversed by sh-JUN co-transfection (Fig. 2I, *P <* 0.01). Overall, TCS-induced c-Jun transcriptionally mediated miR-218-1-3p expression.

### SLC35C1 is a target gene of miR-218-1-3p

**Fig. 3:**
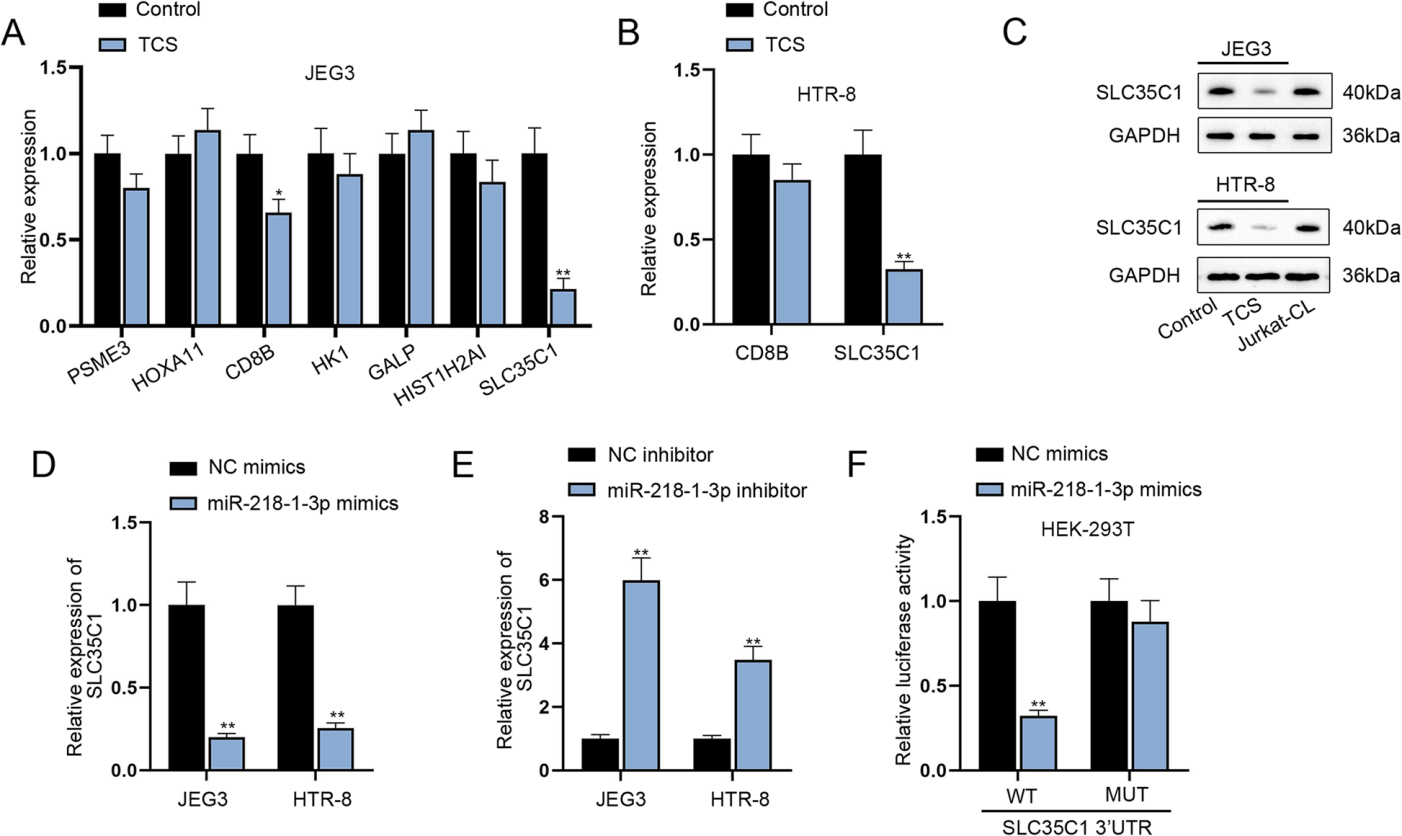
SLC35C1 is a target gene of miR-218-1-3p. **A** RT-qPCR examined the expression of predicted mRNAs in JEG3 cells with or without TCS addition. **B** RT-qPCR detected the expression of CD8B and SLC35C1 in HTR-8 cells. **C** The changes in SLC35C1 protein level were tested in cells with or without TCS treatment. **D-E** SLC35C1 expression was analyzed after transfection of miR-218-1-3p mimics or miR-218-1-3p inhibitor. **F** Luciferase reporter assay validated the binding between miR-218-1-3p and SLC35C1. ^*^P < 0.05, ^**^P < 0.01

To investigate into the downstream mechanism of miR-218-1-3p, TargetScan (http://www.targetscan.org/vert_71/) was applied to predict the possible targets under the condition of Score ≥ 0.8. As a result, a total of 7 target genes (PSME3, HOXA11, CD8B, HK1, GALP, HIST1H2AI and SLC35C1) were predicted. After TCS treatment, only CD8B and SLC35C1 were discovered to be down-regulated in JEG3 cells (Fig. 3A, *P <* 0.05 or *P <* 0.01). CD8B and SLC35C1 expression was then detected in TCS-treated HTR-8 cells, and only decline in SLC35C1 was discovered (Fig. 3B, *P <* 0.01). Therefore, SLC35C1 was selected as the research target. Western blot analysis also uncovered that TCS reduced the protein level of SLC35C1 (Fig. 3C). Additionally, miR-218-1-3p mimics lessened the expression of SLC35C1 while miR-218-1-3p inhibitor enhanced the expression of SLC35C1 (Fig. 3D-E, *P <* 0.01). According to the results of luciferase reporter assay, miR-218-1-3p mimics co-transfection was discovered to notably decrease the luciferase activity of SLC35C1 3’UTR WT group, while that of SLC35C1 3’UTR MUT group was hardly altered under the same condition (Fig. 3F, *P <* 0.01), which suggested the binding correlation between miR-218-1-3p and SLC35C1 at the predicted sites. Taken together, SLC35C1 is targeted and negatively regulated by miR-218-1-3p in trophoblast cells.

### Knockdown of SLC35C1 impedes the proliferation, migration and invasion while accelerating the inflammatory response of trophoblast cells in vitro

**Fig. 4:**
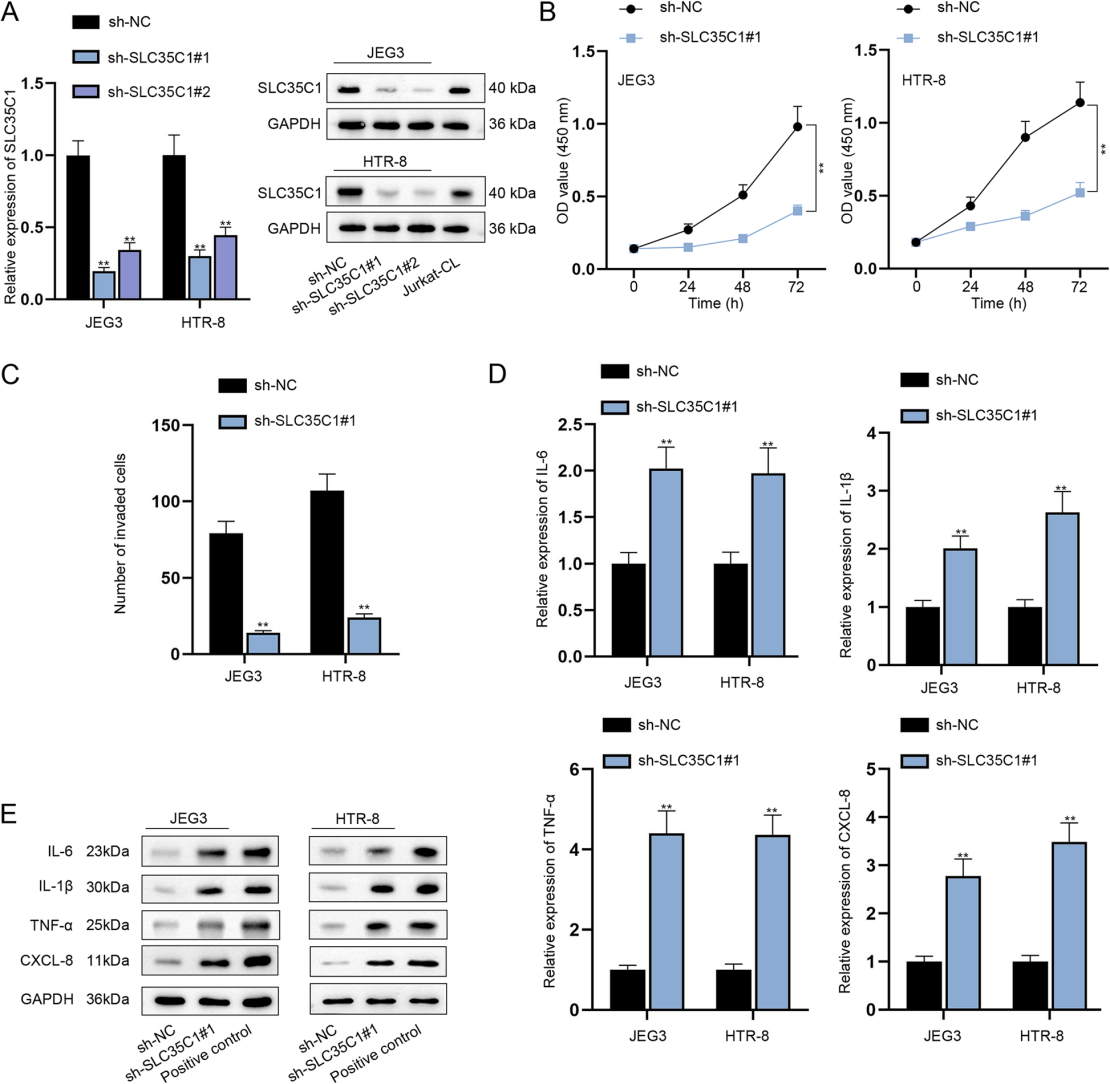
Knockdown of SLC35C1 impedes the proliferation and invasion while accelerating the inflammatory response of trophoblast cells in vitro. **A** The interference efficiency of sh-SLC35C1 was examined. **B-C** The viability and invasion of trophoblast cells were assessed when SLC35C1 was down-regulated. **D-E** RT-qPCR and western blot analyzed the expression of IL-6, IL-1β, TNF-α and CXCL-8 after SLC35C1 was silenced. The samples in the last group of western blot experiment were indicated positive controls for corresponding antibodies. ^**^P < 0.01

To knock down SLC35C1, sh-SLC35C1#1/2 plasmids were transfected into JEG3 and HTR-8 cells and the interference efficiency was tested to be high (Fig. 4A, *P <* 0.01). Also, sh-SLC35C1#1 was chosen for following functional assays for its higher knockdown efficiency. CCK-8, wound healing and transwell assays proved that depletion of SLC35C1 weakened the proliferative, migratory and invasive abilities of trophoblast cells (Fig. 4B-C& Supplementary Fig. 1 J, *P <* 0.01). Additionally, RT-qPCR and western blot analysis indicated that SLC35C1 inhibition motivated the inflammatory response of JEG3 and HTR-8 cells as a notable augment in mRNA and protein levels of the proinflammatory factors was observed after sh-SLC35C1#1 transfection (Fig. 4D-E, *P <* 0.01). To be summarized, SLC35C1 aggravates the proliferation, migration and invasion while alleviating the inflammatory response of trophoblast cells in vitro.

### TCS suppresses the proliferation, migration and invasion while inducing the inflammatory response of trophoblast cells via modulation of miR-218-1-3p/SLC35C1 axis

**Fig. 5:**
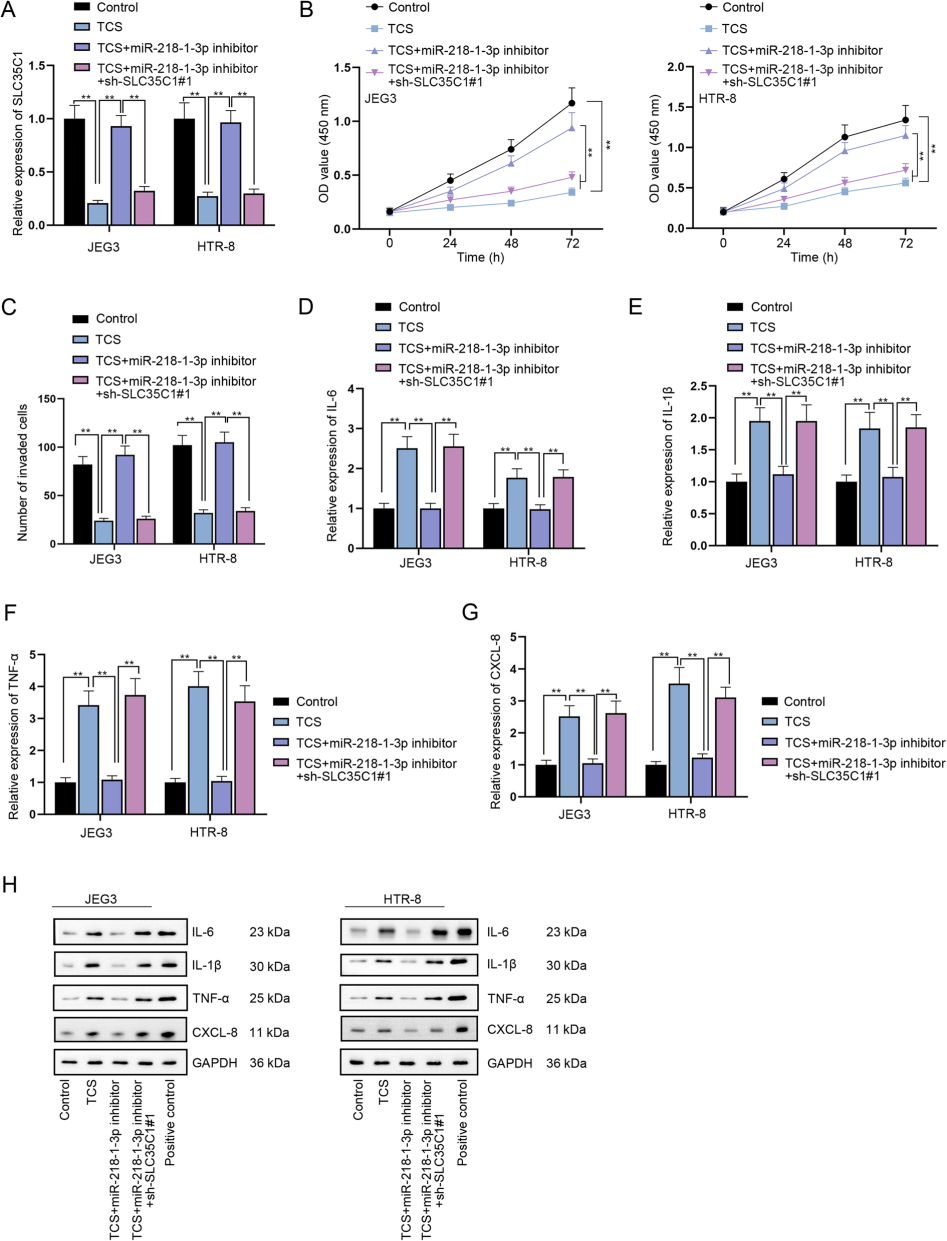
TCS suppresses the proliferation and invasion while inducing the inflammatory response of trophoblast cells in vitro via modulation of miR-218-1-3p/SLC35C1 axis. **A** SLC35C1 expression was tested by RT-qPCR in the Control group, TCS group, TCS + miR-218-1-3p inhibitor group and TCS + miR-218-1-3p inhibitor+sh-SLC35C1#1 group. **B-C** Cell proliferation and invasion were evaluated in different groups via CCK-8 and transwell assays. **D-H** RT-qPCR and western blot analyzed the expression of IL-6, IL-1β, TNF-α and CXCL-8 in different groups. The samples in the last group of western blot experiment were indicated positive controls for corresponding antibodies. ^**^P < 0.01

To determine the validity of TCS/miR-218-1-3p/SLC35C1 axis in trophoblast cells, rescue assays were carried out. RT-qPCR outcomes suggested that the decline in SLC35C1 expression caused by TCS addition was reversed by miR-218-1-3p inhibitor transfection, which was again reduced by SLC35C1 down-regulation (Fig. 5A, *P <* 0.01). Through CCK-8, wound healing and transwell assays, it was evidenced that miR-218-1-3p inhibition could countervail the suppressive influences of TCS treatment on cell proliferative, migratory and invasive abilities, but SLC35C1 deficiency could completely counteract the effects of miR-218-1-3p inhibition (Fig. 5B-C & Supplementary Fig. 1 K, *P <* 0.01). Moreover, the promoted inflammatory response of JEG3 and HTR-8 cells treated with TCS was attenuated by miR-218-1-3p inhibition and this influence was offset by SLC35C1 knockdown (Fig. 5D-H, *P <* 0.01). Collectively, TCS mediated miR-218-1-3p/SLC35C1 axis can influence trophoblast cell proliferation, migration, invasion and inflammatory response in vitro.

## Discussion

Spontaneous abortion is a serious problem troubling women at the reproductive age [1]. The trophoblast has been reported as a crucial effector in implantation and placentation [23]. Wang et al. have reported the association between TCS and spontaneous abortion in human cases and mice model [13]. Aiming to explore the underlying regulatory mechanism, we firstly utilized TCS to create in vitro model simulating spontaneous abortion in trophoblast cells and discovered that TCS suppressed cell proliferation, migration and invasion while inducing the inflammatory response of trophoblast cells in vitro.

SLIT2 has been identified as an important regulator in trophoblast differentiation and invasion during pregnancy [20]. It has been reported that Benzophenone-3 (BP-3) treatment can mediate the expression of SLIT2 and miR-218 [24], a gene derived from introns of SLIT2 [21]. Moreover, miR-218 has also been reported to play a central part in regulating trophoblast functions. For instance, hypoxia-induced miR-218 exerts suppressive impacts on trophoblast invasion via targeting LASP1 [25]. Additionally, miR-218 weakens the migratory and invasive abilities of HTR-8 cells via targeting SOX4 [26]. In our study, we discovered that TCS induced the up-regulation of miR-218-1-3p in human trophoblast cells. It was suggested in functional assays that overexpression of miR-218-1-3p impeded the proliferation, migration and invasion while stimulating the inflammatory response of trophoblast cells.

The involvement of c-Jun in multiple human diseases has also been unveiled in previous papers. For example, Peng et al. have illustrated that c-Jun, mediated by GnRH, influences trophoblast cell invasion [27]. C-Jun also displays peak expression in human placenta at early gestation, which might be associated with cytotrophoblastic proliferation [28]. As a transcription factor, c-Jun also influences gene transcription. For example, c-Jun restrains the transcriptional activity of NF-E2 via inactive c-Jun/NF-E2p18 heterocomplexes [29]. Additionally, TCS has been mentioned to modulate c-Jun production [22]. Through our investigation, we discovered that TCS at a high concentration also contributed to an augment in c-Jun level. Moreover, c-Jun activated the transcription of MIR218–1 to enhance the expression of miR-218-1-3p.

SLC35C1 has been uncovered to abrogate the progression of colon cancer via inactivation of Wnt pathway [30]. Our experimental results confirmed that SLC35C1 was a target gene of miR-218-1-3p, and the negative correlation between SLC35C1 and miR-218-1-3p was ascertained. In addition, SLC35C1 silence hindered the proliferation, migration and invasion of trophoblast cells while promoting cellular inflammatory response. Rescue assays also validated that TCS modulated the proliferation, migration, invasion and inflammatory response of trophoblast cells in vitro via miR-218-1-3p/SLC35C1 axis.

To be summarized, miR-218-1-3p was testified to be highly expressed in trophoblast cells after TCS treatment and up-regulation of miR-218-1-3p impeded the proliferation, migration and invasion of trophoblast cells while stimulating cellular inflammatory response in vitro. Moreover, TCS-induced c-Jun could activate the transcription of MIR218–1, thereby enhancing miR-218-1-3p expression. After confirming SLC35C1 was targeted by miR-218-1-3p, we also confirmed the influences of miR-218-1-3p/SLC35C1 on TCS-medicated proliferation, migration, invasion and inflammation of trophoblast cells. In a word, our study unveiled the TCS/miR-218-1-3p/SLC35C1 axis in the modulation of trophoblast cells and might provide novel insights for preventing spontaneous abortion in vitro.

However, there are still some limitations in our present study. For example, whether other potential regulatory mechanisms in the downstream of TCS participate in the process of spontaneous abortion also needs to be confirmed. This study used “in vitro” methodology to evaluate the mechanism of action of TCS in the trophoblast. The association of TCS with abortion in humans needs further studies.

## Supplementary Information

**Additional file 1: Supplementary Fig. 1.** (A) CCK-8 assay examined cell viability under treatment with different concentrations of TCS. (B) Wound healing assay assessed cell migration under TCS addition. (C) Transwell assay evaluated the invasive capacity of trophoblast cells with TCS treatment. (D-H) RT-qPCR and western blot analyzed the expression of proinflammatory factors under the treatment of TCS. The samples in the last group of western blot experiment were indicated positive controls for corresponding antibodies. (I-J) Wound healing assay assessed cell migration under the influence of miR-218-1-3p overexpression or SLC35C1 knockdown. (K) Cell migration under transfection of indicated plasmids was evaluated via wound healing assay. ^**^P < 0.01.

**Additional file 2: Supplementary file 1.** Morphology images of JEG3 and HTR-8 cells.

**Additional file 3: Supplementary file 2.** STR reports of JEG3 cells.

**Additional file 4: Supplementary file 3.** STR reports of HTR-8 cells.

**Additional file 5: Supplementary file 4.** Details of Ct values in real-time PCR analyses.

**Additional file 6: Supplementary file 5.** Original western blots.

## Data Availability

All data and material used and/or analyzed during the current study are available from the corresponding authors upon reasonable request.
