# Supplementary figures and images for: Triclosan activates c-Jun/miR-218-1-3p/SLC35C1 signaling to regulate cell viability, migration, invasion and inflammatory response of trophoblast cells in vitro
Source: BMC Pregnancy Childbirth. 2022 Jun 6;22:470. doi: 10.1186/s12884-022-04791-z (PMC9172191; doi:10.1186/s12884-022-04791-z)

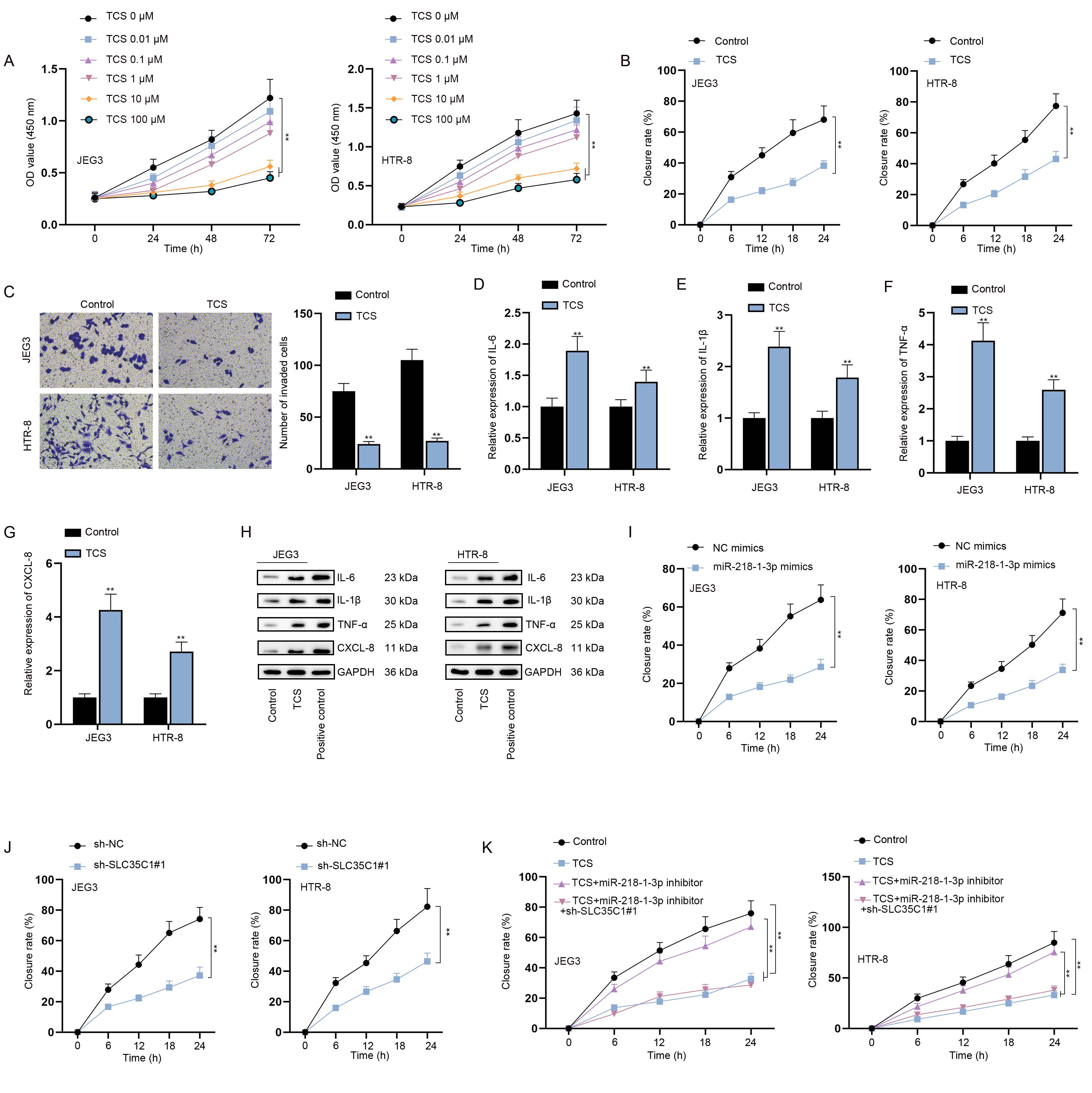

Supplement: Supplementary file 1 — Additional file 1: Supplementary Fig. 1. (A) CCK-8 assay examined cell viability under treatment with different concentrations of TCS. (B) Wound healing assay assessed cell migration under TCS addition. (C) Transwell assay evaluated the invasive capacity of trophoblast cells with TCS treatment. (D-H) RT-qPCR and western blot analyzed the expression of proinflammatory factors under the treatment of TCS. The samples in the last group of western blot experiment were indicated positive controls for corresponding antibodies. (I-J) Wound healing assay assessed cell migration under the influence of miR-218-1-3p overexpression or SLC35C1 knockdown. (K) Cell migration under transfection of indicated plasmids was evaluated via wound healing assay. **P < 0.01. [file 12884_2022_4791_MOESM1_ESM.tif]

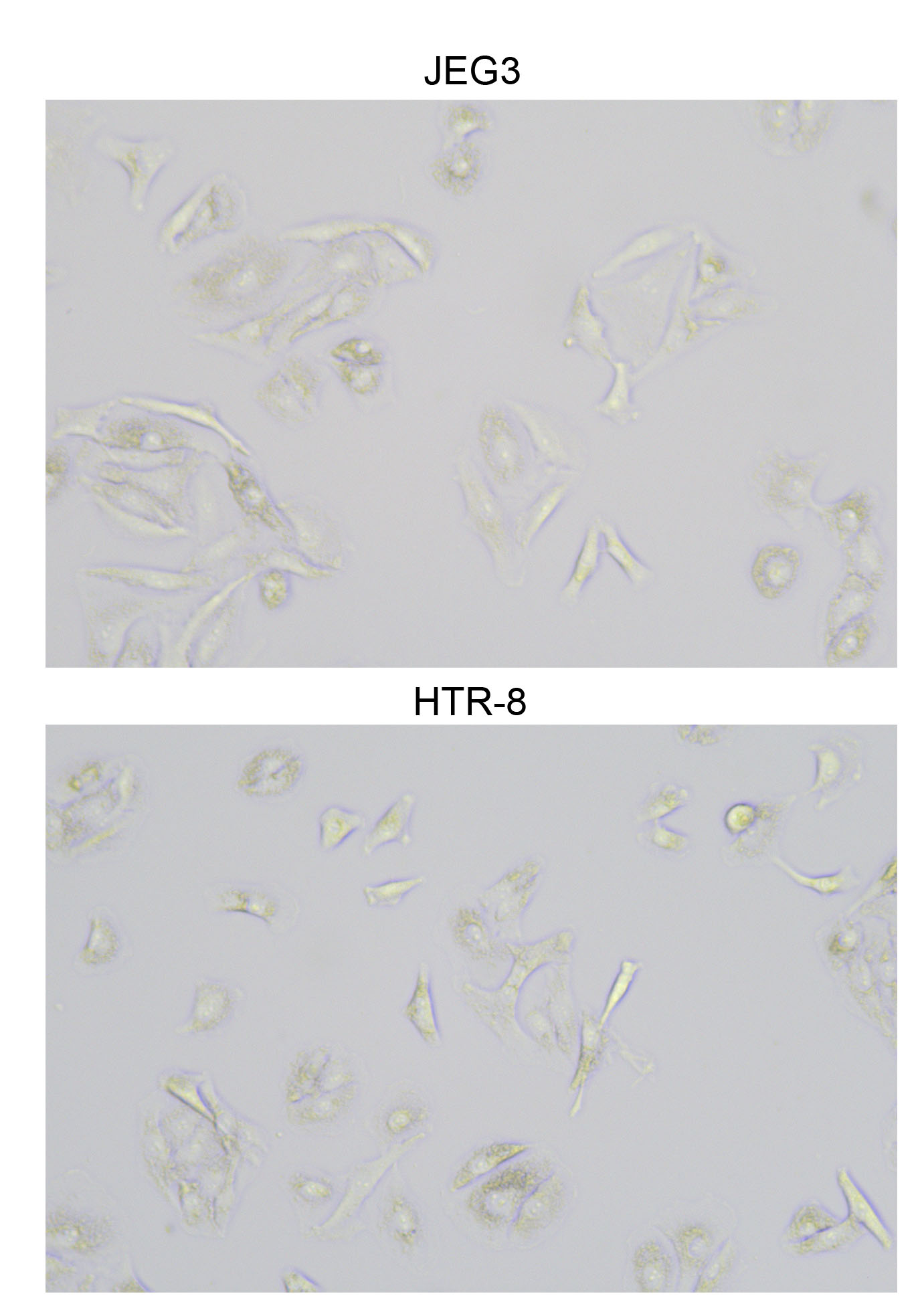

Supplement: Supplementary file 2 — Additional file 2: Supplementary file 1. Morphology images of JEG3 and HTR-8 cells. [file 12884_2022_4791_MOESM2_ESM.jpg]

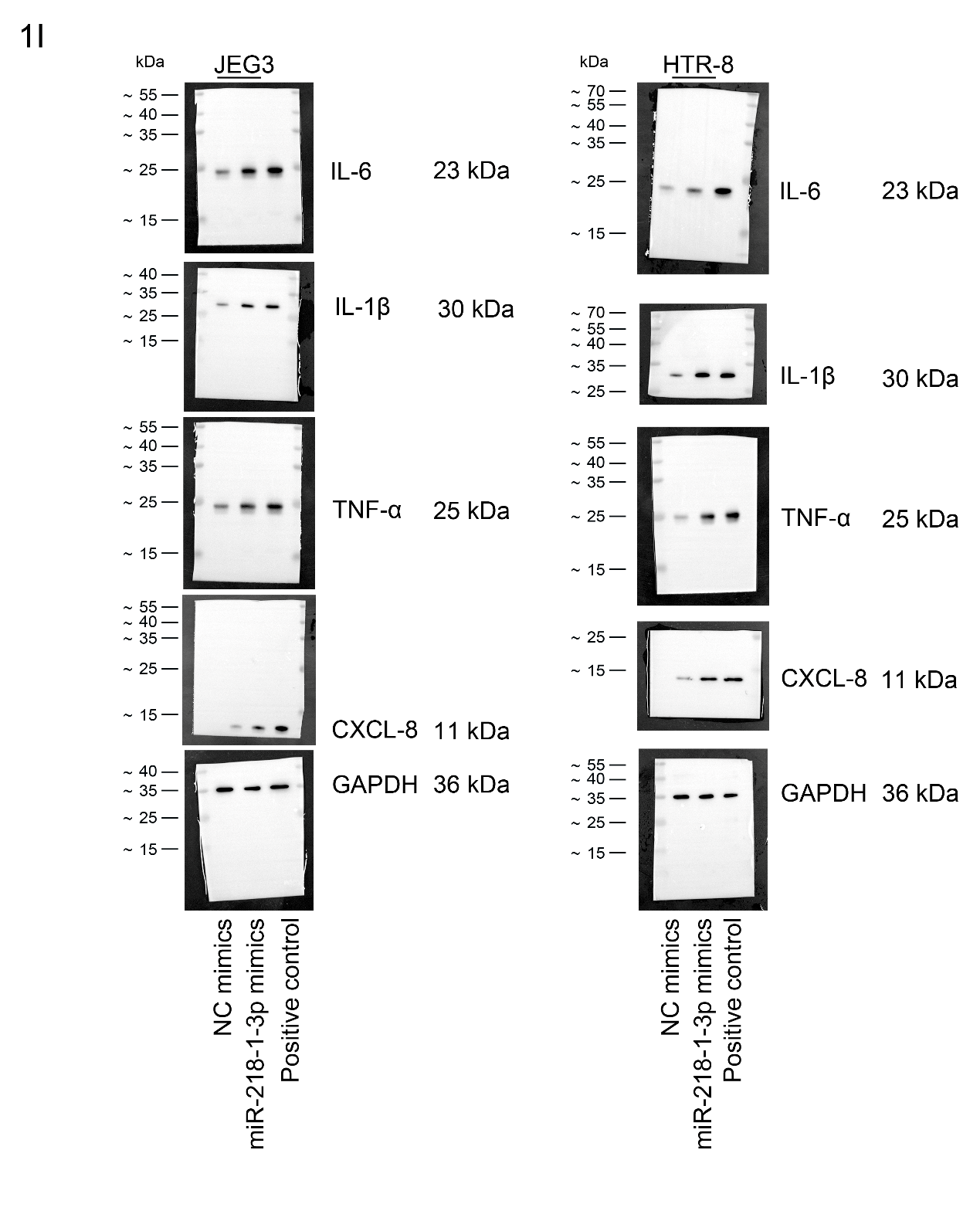


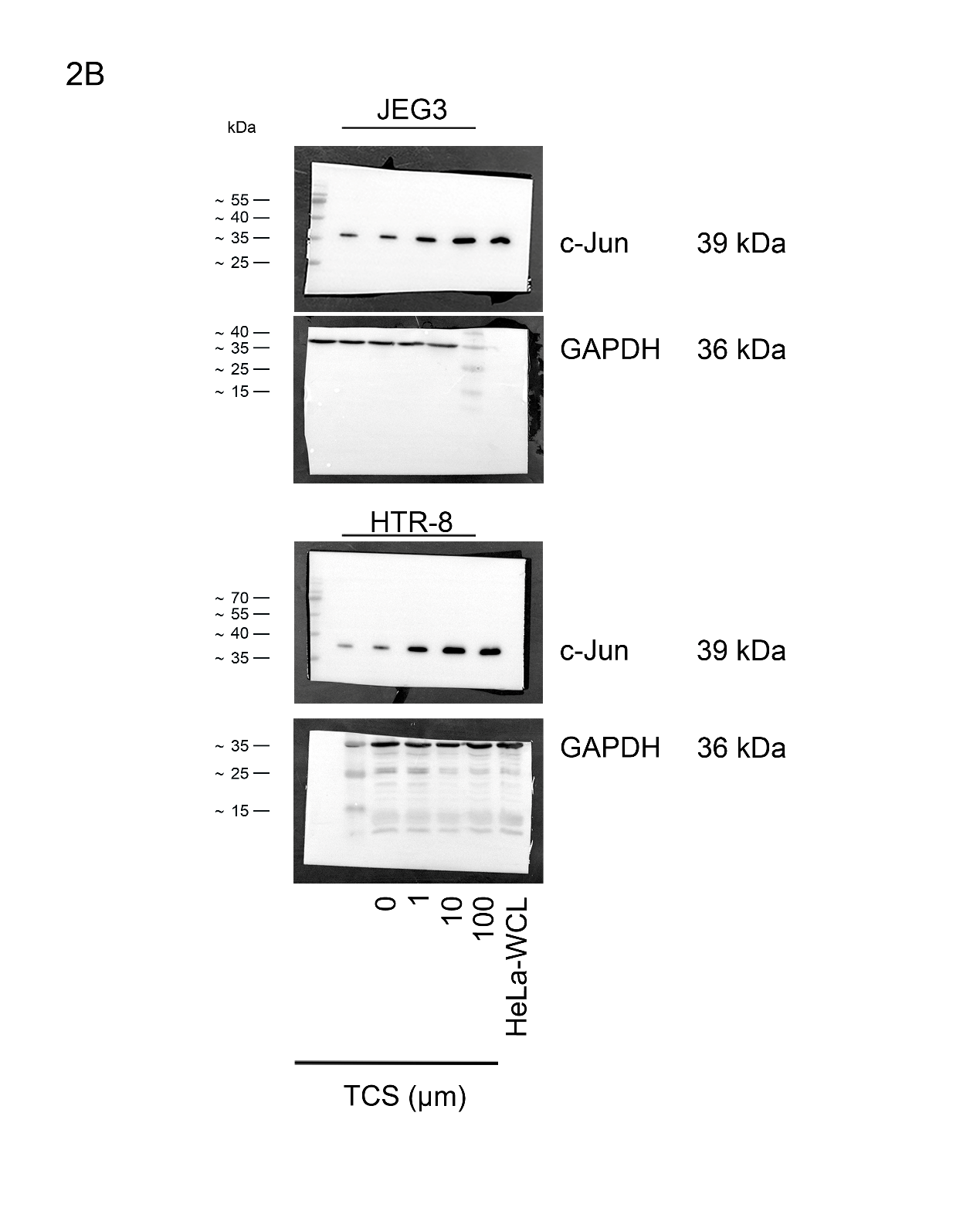


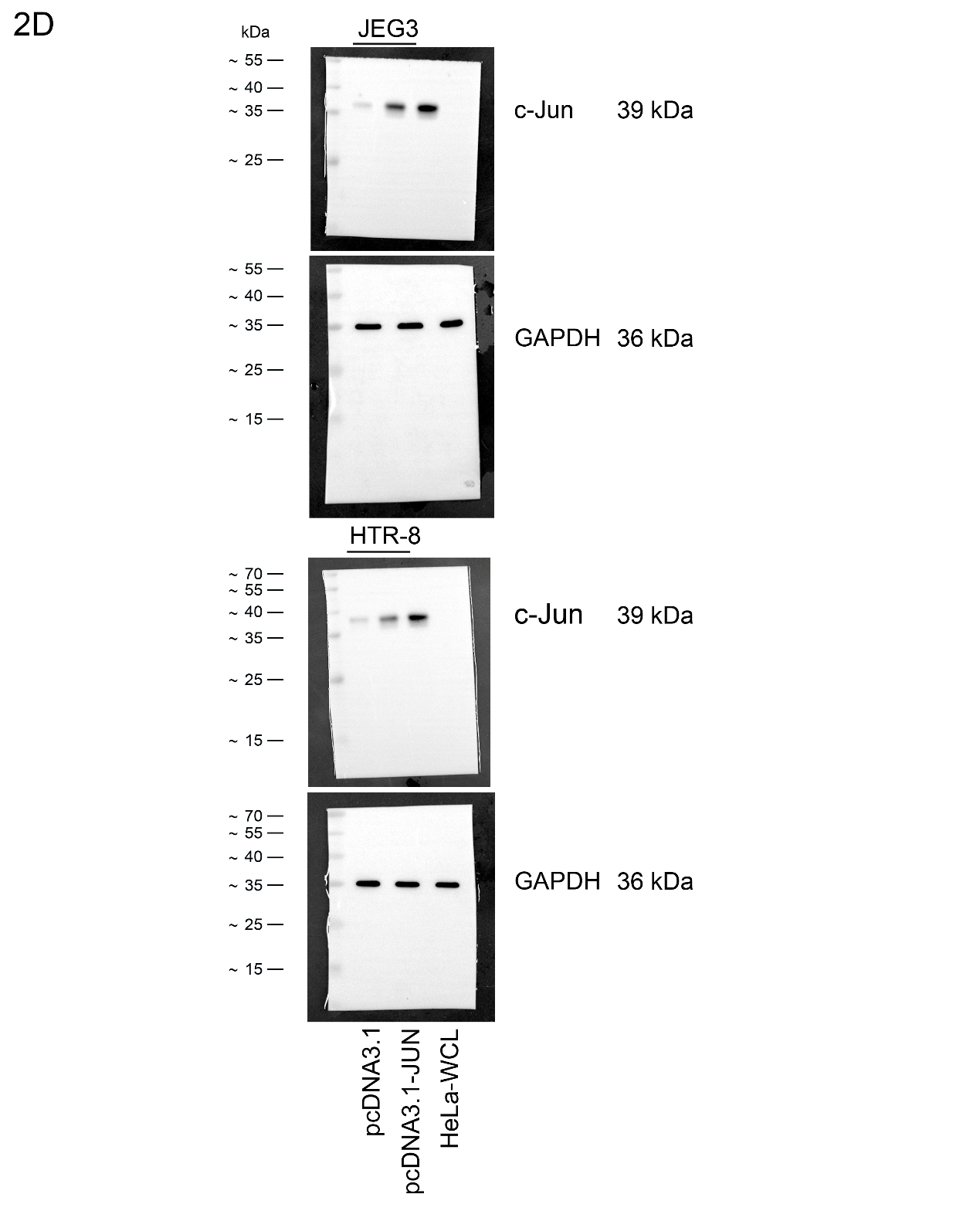


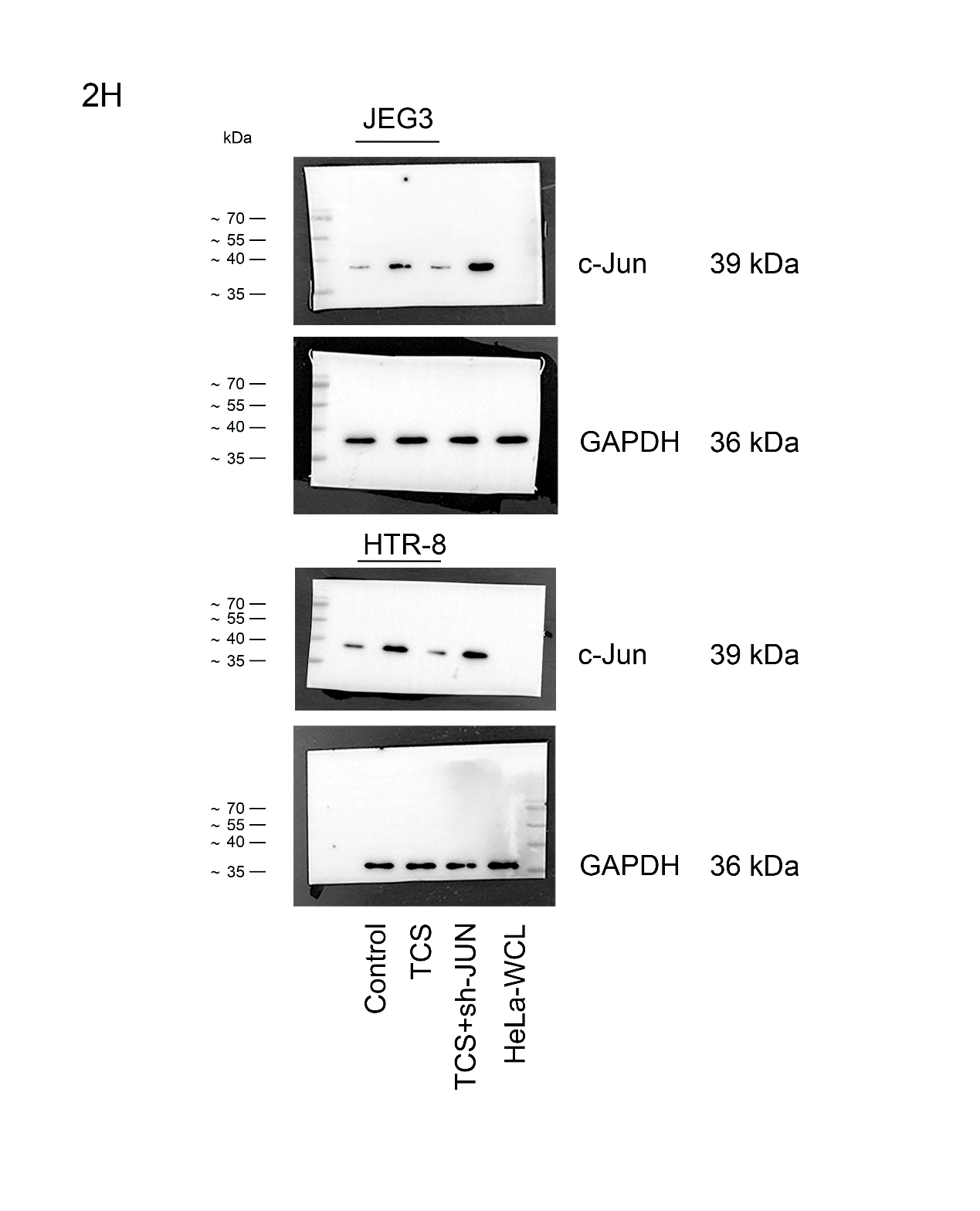


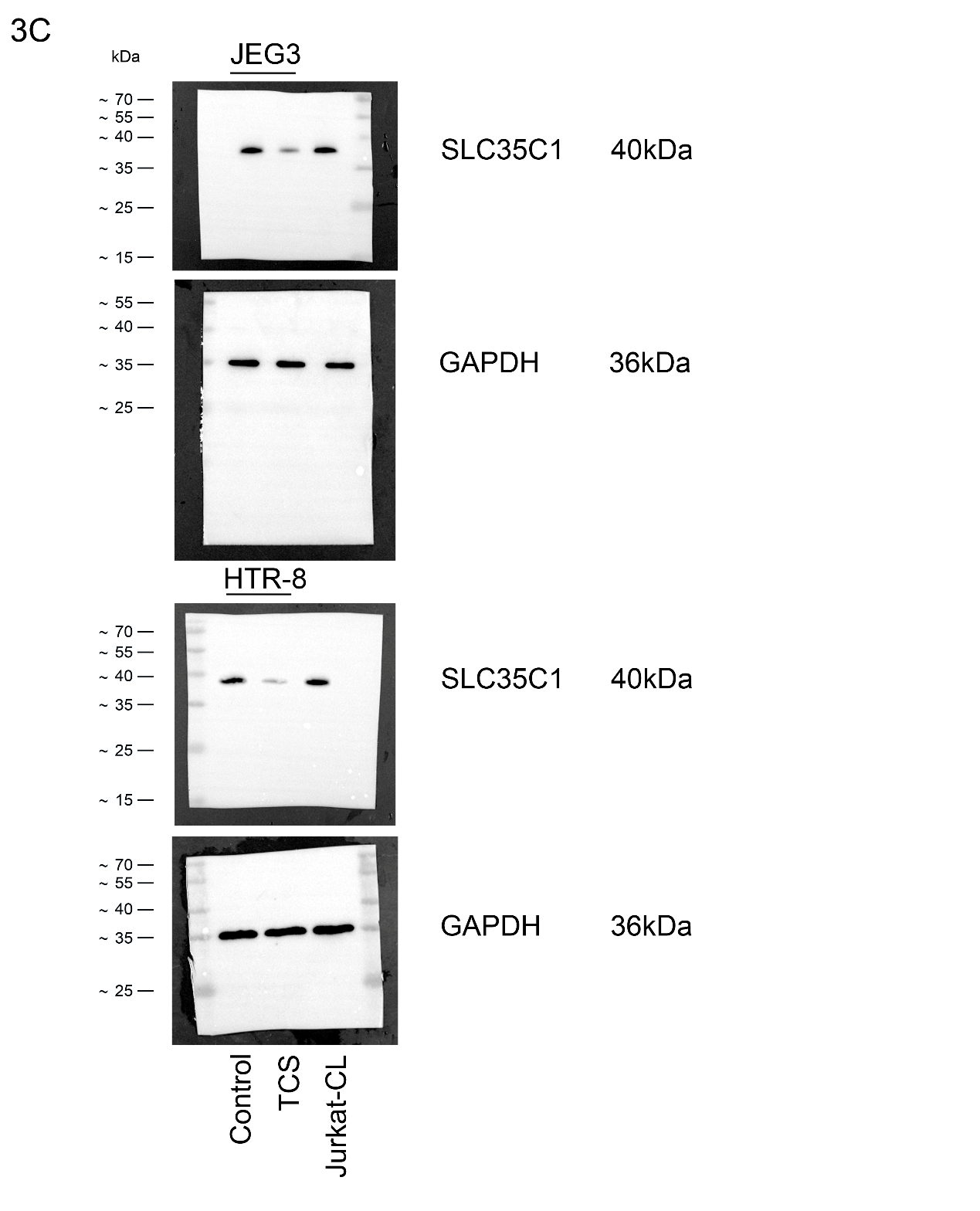


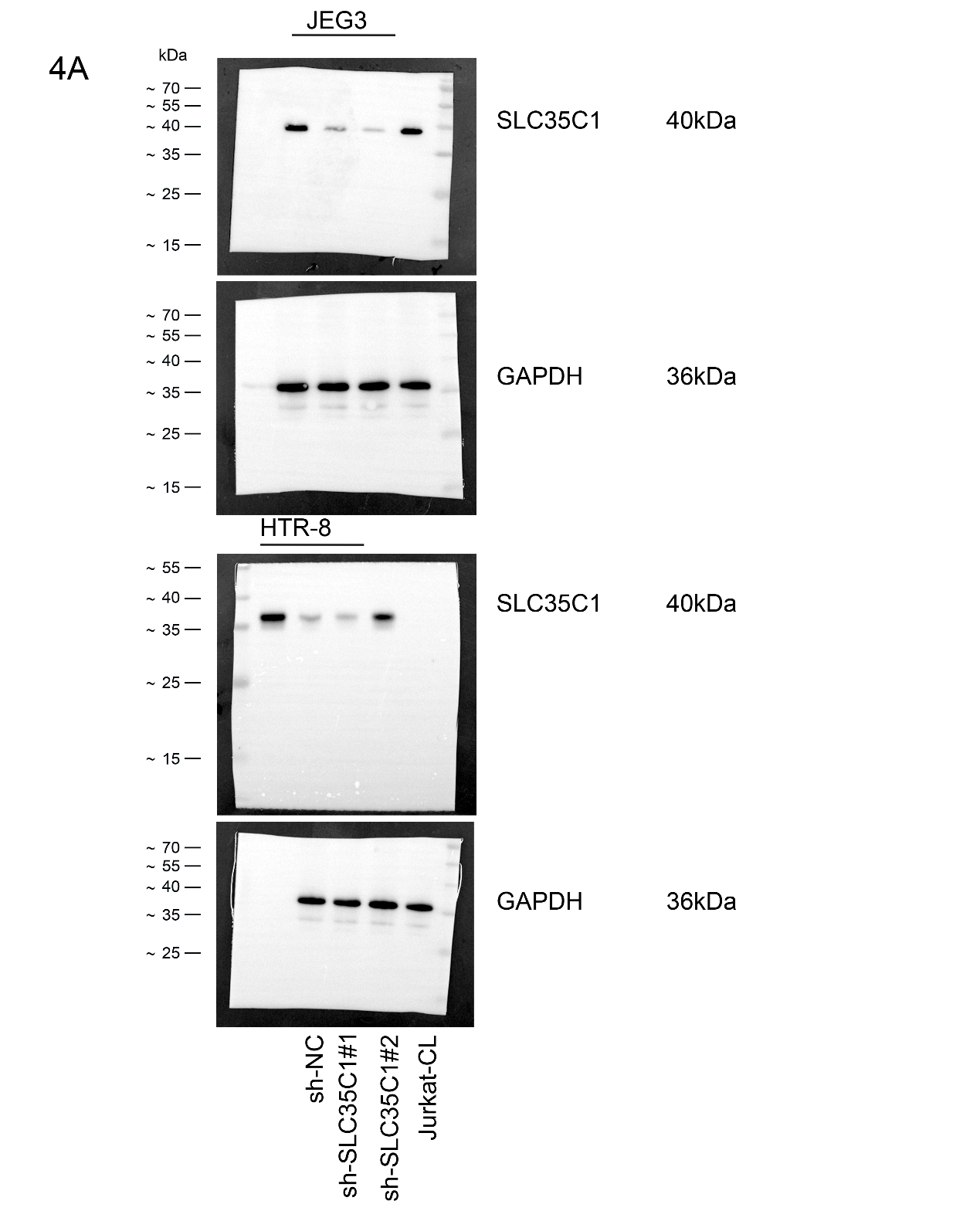


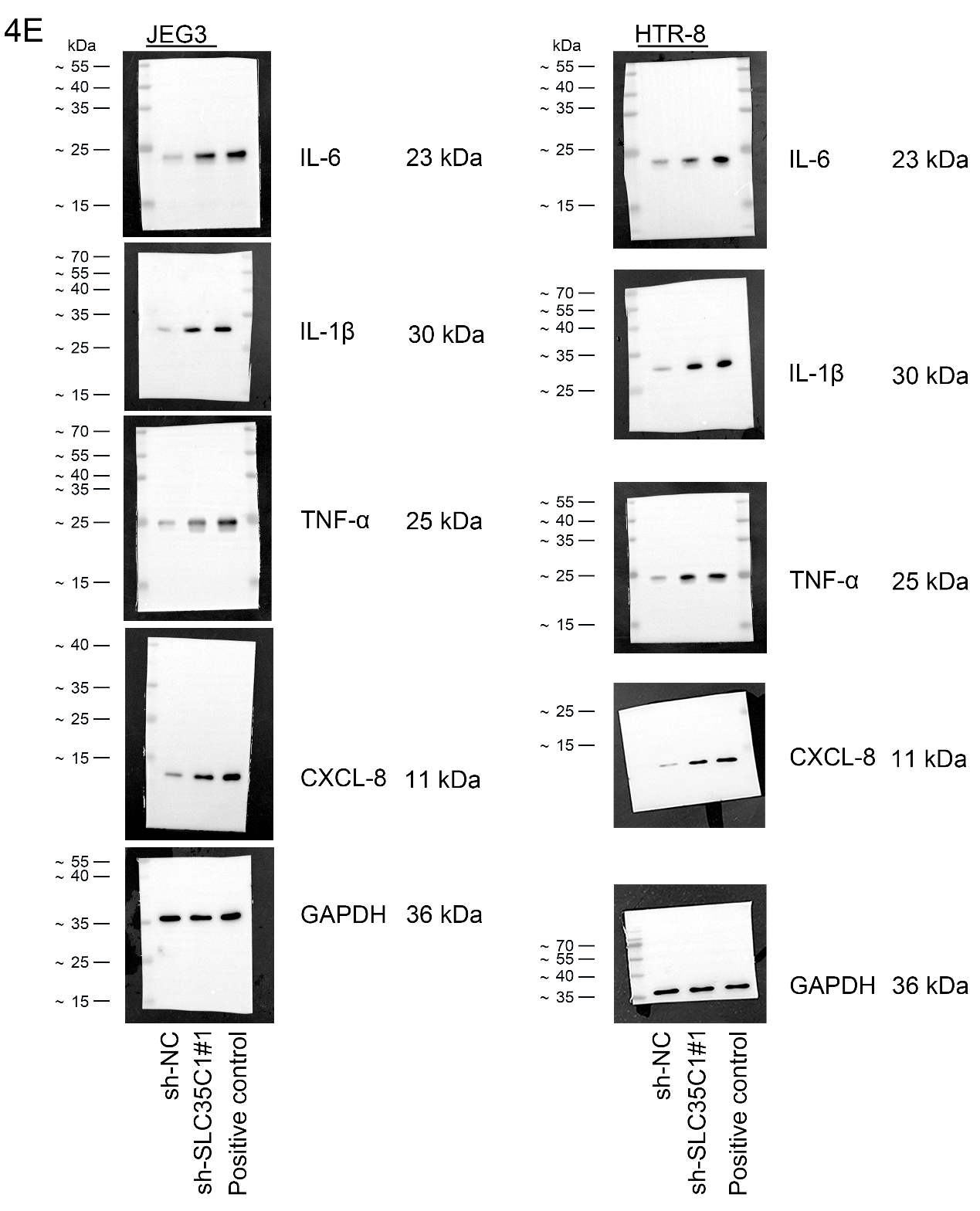


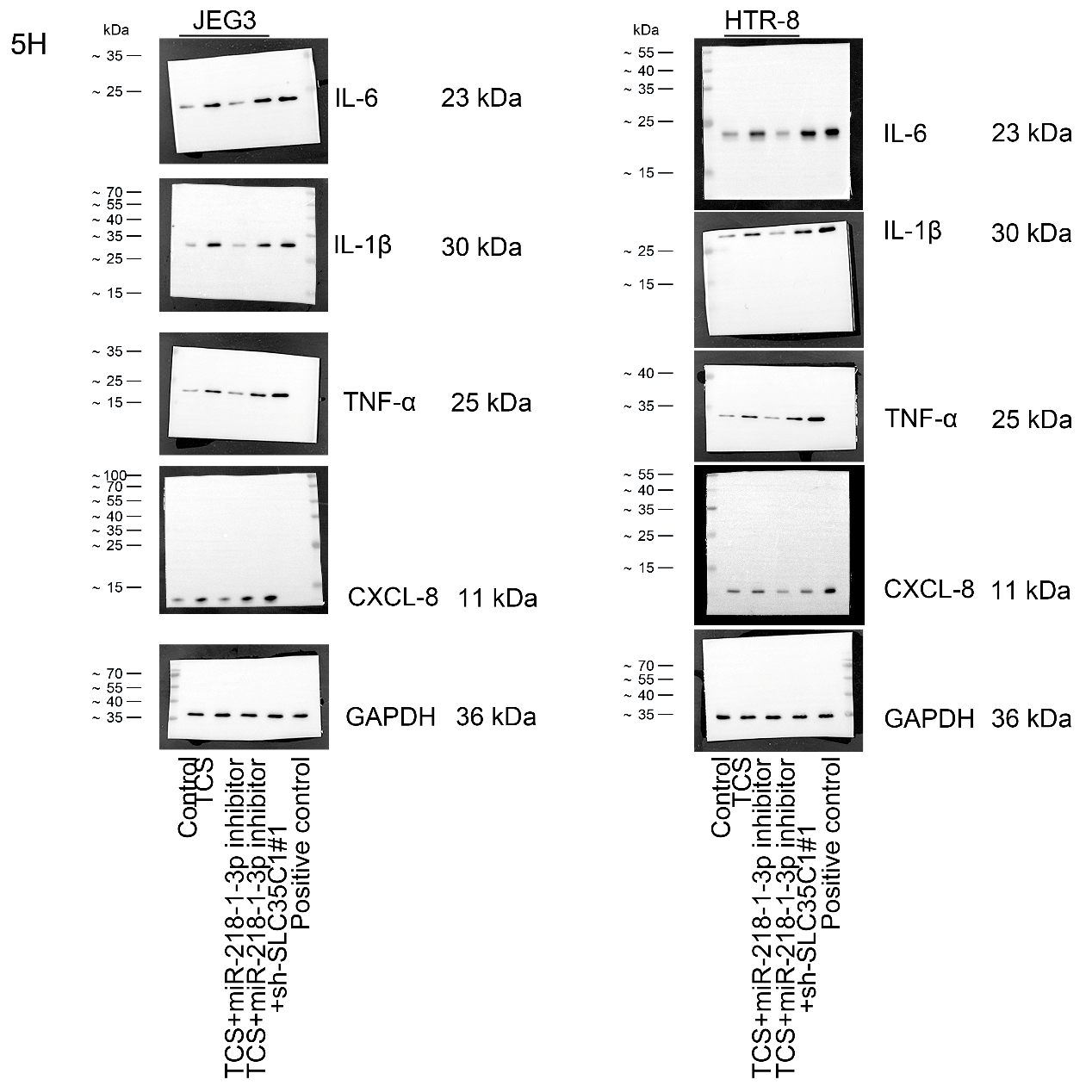


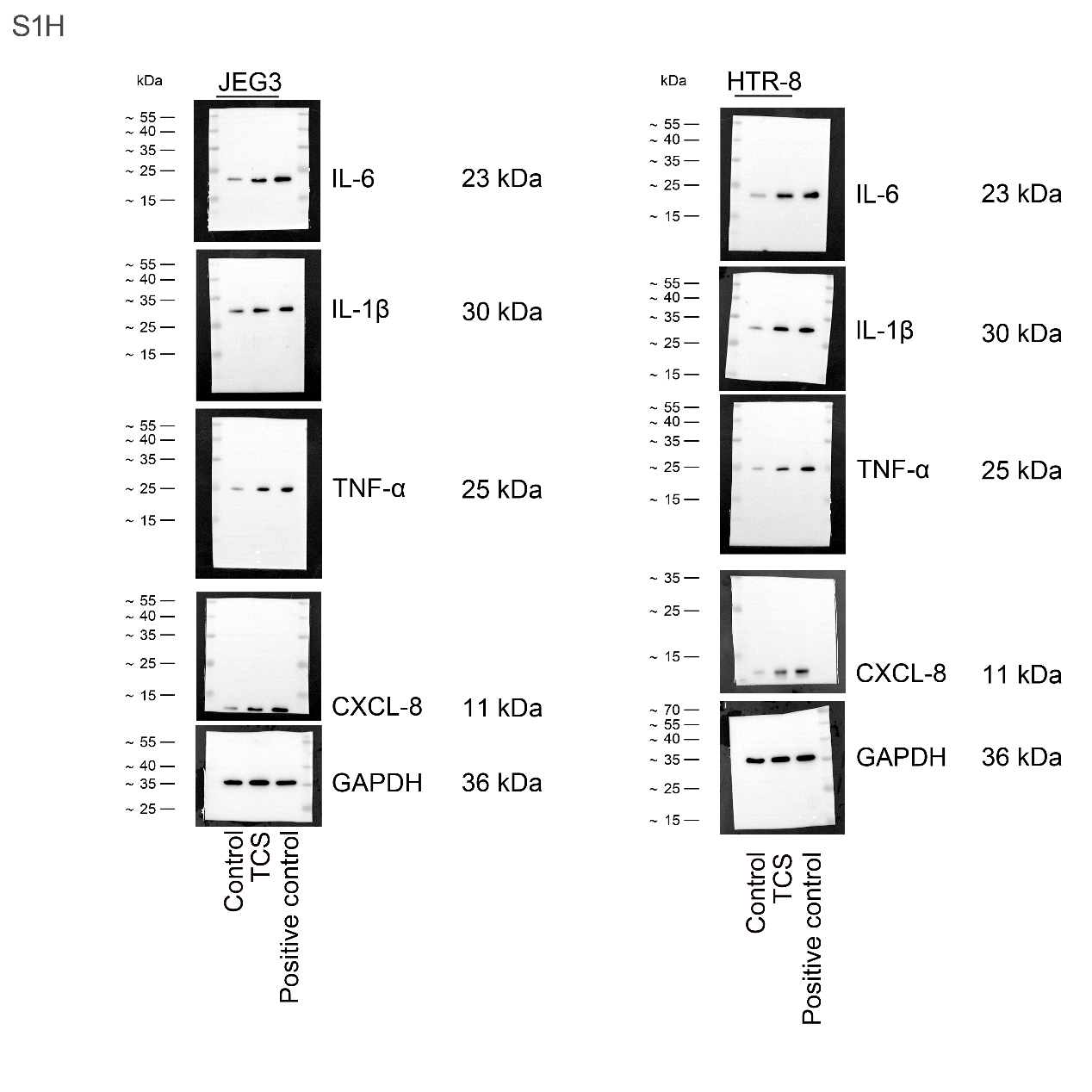

Supplement: Supplementary file 6 — Additional file 6: Supplementary file 5. Original western blots. [file 12884_2022_4791_MOESM6_ESM.docx]
