# Supplementary material for: Triclosan activates c-Jun/miR-218-1-3p/SLC35C1 signaling to regulate cell viability, migration, invasion and inflammatory response of trophoblast cells in vitro
Source: BMC Pregnancy Childbirth. 2022 Jun 6;22:470. doi: 10.1186/s12884-022-04791-z (PMC9172191; doi:10.1186/s12884-022-04791-z)
Supplement: Supplementary file 3 — Additional file 3: Supplementary file 2. STR reports of JEG3 cells. [file 12884_2022_4791_MOESM3_ESM.pdf]

细胞遗传质量鉴定检验报告

检品名称：细胞系

检验类型：STR 基因型检验

样品编号：

表 1 样本编号

客户样本编号	公司编号
125	20170221-07

样品数量：1

样品性状：细胞系

检测项目：STR

送检单位：上海富衡生物科技有限公司

检测方法：用 Axygen 的基因组抽提试剂盒提取 DNA，采用 20- STR 扩增方案扩增，在 ABI 3730XL 型遗传分析仪上对 STR 位点和性别基因 Amelogenin 进行检测。

检验结果：

(一) 检验基本情况

表 2：样本基因型检验结果

	**多等位基因**	**匹配细胞系**	**细胞库**	**EV 值**	**匹配说明**
20170221-07	无	JEG-3 等	DSMZ	1	完全匹配

- 多等位基因指三等位及以上基因现象。

- 本次检测各细胞分型结果良好。

(二) 各样本描述

- 20170221-07：该株细胞 DNA 分型在细胞系检索中找到**完全匹配**的细胞系，DSMZ 数据库显示细胞名为 **JEG-3 等**，细胞号对应 **463 等**。本次检测在该细胞系中**没有发现多等位基因**。

(三) 样本分型结果

表 3：细胞 20170221-07 的 STR 位点和 Amelogenin 位点的基因分型结果

Marker	样本				细胞库信息		
	Allele1	Allele2	Allele3	Allele4	Allele1	Allele2	Allele3
D5S818	10	11			10	11	
D13S317	9	11			9	11	
D7S820	10	12			10	12	
D16S539	13	14			13	14	
VWA	16	16			16	16	
TH01	9	9.3			9	9.3	
AMEL	X	Y			X	Y	
TPOX	8	8			8	8	
CSF1PO	11	12			11	12	
D12S391	17	24					
FGA	23	24					
D2S1338	24	24					
D21S11	30	30					
D18S51	14	14					
D8S1179	12	12					
D3S1358	15	16					
D6S1043	11	11					
PENTAE	8	12					
D19S433	13	15					
PENTAD	9	12					

其他说明：

(一) 分型方案及位点分布：

附表：实验方案及位点

	方案 1	方案 2	方案 3	方案 4
1	TH01	TPOX	D3S1358	AMEL
2	D12S391	VWA	D13S317	D5S818
3	D7S820	D8S1179	D6S1043	D2S1338
4	CSF1PO	PENTAD	D16S539	D21S11
5	FGA		D19S433	D18S51
6	PENTAE			

Applied Biosystems
GeneMapper 4.0

Cell Line Authenticaion-10

Sample File	Sample Name	Panel	OS	SQ
31_G04_CellLineAuthentication-0223.fsa	125	STR Profile 1-human		

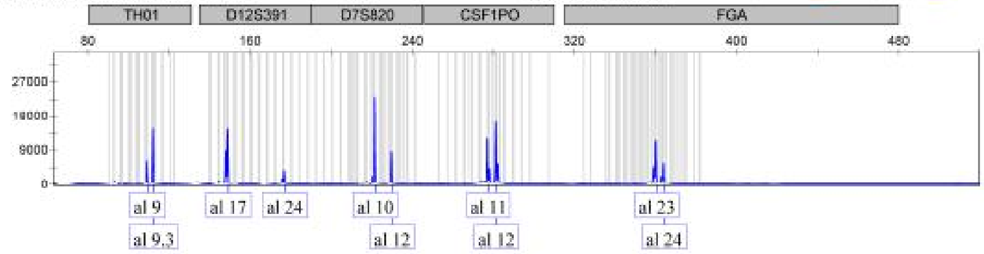

31_G04_CellLineAuthentication-0223.fsa	125	STR Profile 1-human		

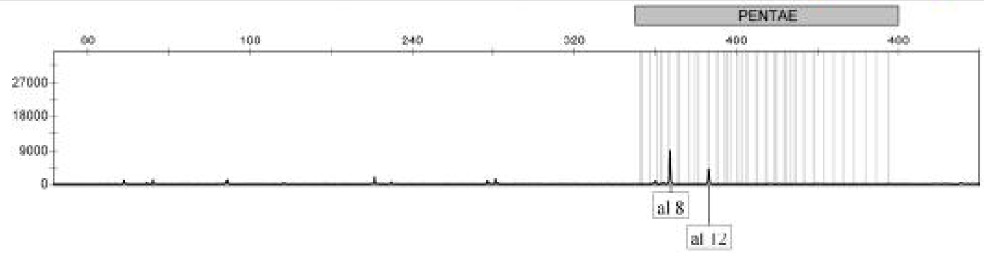

55_G07_CellLineAuthentication-0223.fsa	125	STR Profile 2-human		

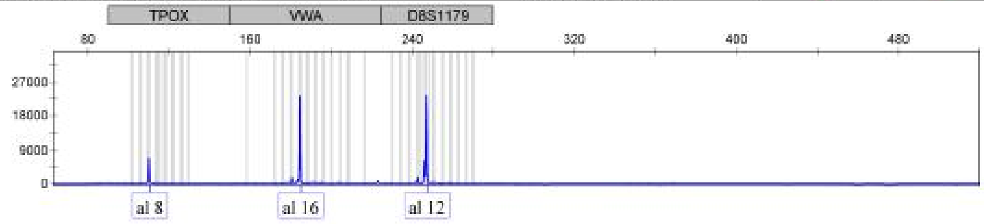

Sample File	Sample Name	Panel	OS	SQ
55_G07_CellLineAuthentication-0223.fsa	125	STR Profile 2-human		

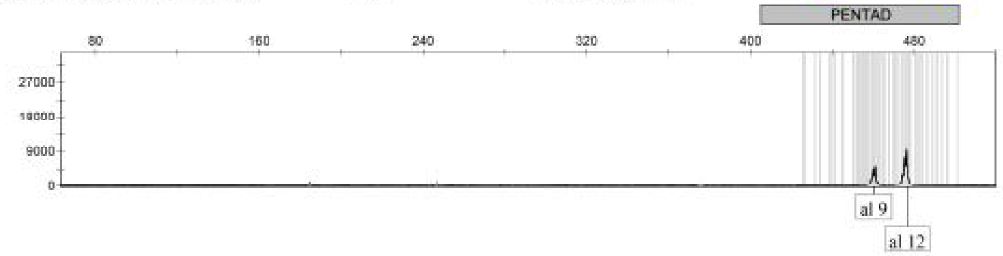

Sample File	Sample Name	Panel	OS	SQ
79_G10_CellLineAuthentication-0223.fsa	125	STR Profile 3-human		

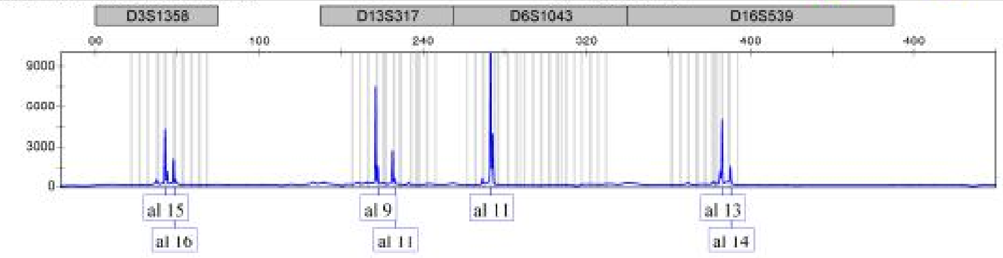

Sample File	Sample Name	Panel	OS	SQ
79_G10_CellLineAuthentication-0223.fsa	125	STR Profile 3-human		

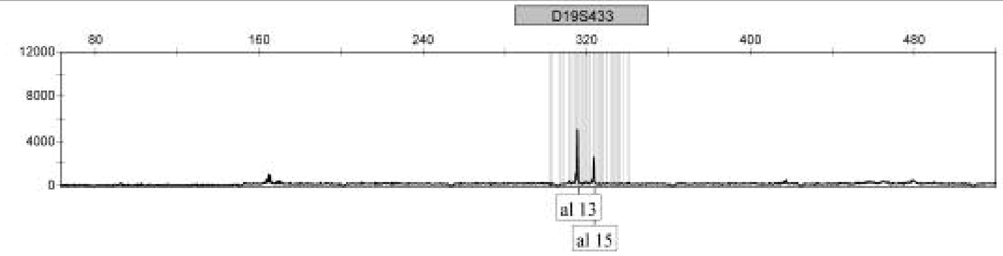

Applied Biosystems
GeneMapper 4.0

Cell Line Authenticaion-10

Sample File	Sample Name	Panel	OS	SQ
07_G01_CellLineAuthentication-0223.fsa	125	STR Profile 4-human		

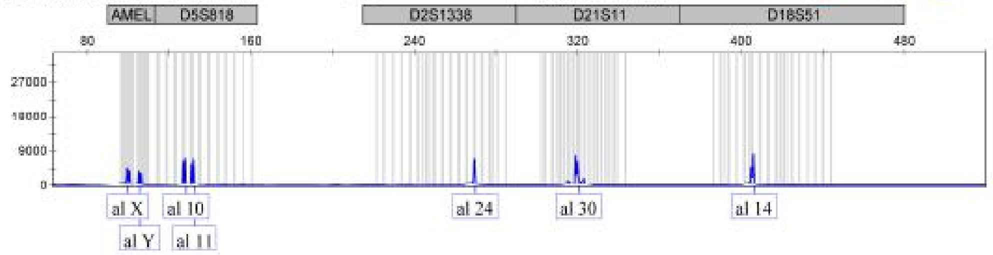

07_G01_CellLineAuthentication-0223.fsa 125 STR Profile 4-human

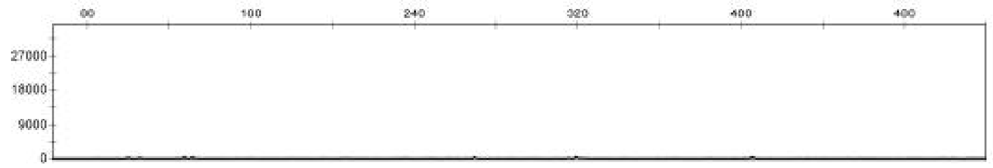

签发日期：
2017 年 03 月
