# Supplementary material for: Triclosan activates c-Jun/miR-218-1-3p/SLC35C1 signaling to regulate cell viability, migration, invasion and inflammatory response of trophoblast cells in vitro
Source: BMC Pregnancy Childbirth. 2022 Jun 6;22:470. doi: 10.1186/s12884-022-04791-z (PMC9172191; doi:10.1186/s12884-022-04791-z)
Supplement: Supplementary file 5 — Additional file 5: Supplementary file 4. Details of Ct values in real-time PCR analyses. [file 12884_2022_4791_MOESM5_ESM.pdf]

**Supplementary file 4: CT values for in PCR.**

| Figure | Cell | Plasmid transfection/cell treatment | Gene | CT values | | |
|---|---|---|---|---|---|---|
| Fig. 1A | JEG3 | Control | miR-218-1-3p | 27.53 | 27.73 | 27.79 |
| | HTR-8 | Control | miR-218-1-3p | 26.48 | 26.49 | 26.71 |
| | JEG3 | TCS | miR-218-1-3p | 25.43 | 25.6 | 25.24 |
| | HTR-8 | TCS | miR-218-1-3p | 23.48 | 23.1 | 23.36 |
| | JEG3 | Control | U6 | 18.89 | 18.96 | 19.47 |
| | HTR-8 | Control | U6 | 18.05 | 18.11 | 18.46 |
| | JEG3 | TCS | U6 | 19.61 | 18.65 | 18.68 |
| | HTR-8 | TCS | U6 | 18 | 17.74 | 17.71 |
| Fig. 1B | JEG3 | NC mimics | miR-218-1-3p | 27.75 | 27.45 | 27.66 |
| | HTR-8 | NC mimics | miR-218-1-3p | 26.21 | 26.21 | 26.41 |
| | JEG3 | miR-218-1-3p mimics | miR-218-1-3p | 25.05 | 24.84 | 24.71 |
| | HTR-8 | miR-218-1-3p mimics | miR-218-1-3p | 23.85 | 23.43 | 23.54 |
| | JEG3 | NC mimics | U6 | 19.02 | 19.36 | 18.94 |
| | HTR-8 | NC mimics | U6 | 18.38 | 18.03 | 17.51 |
| | JEG3 | miR-218-1-3p mimics | U6 | 18.8 | 19.09 | 18.66 |
| | HTR-8 | miR-218-1-3p mimics | U6 | 17.53 | 17.81 | 17.58 |
| Fig. 1E | JEG3 | NC mimics | IL-6 | 23.34 | 23.36 | 23.84 |
| | HTR-8 | NC mimics | IL-6 | 23.18 | 23.52 | 23.21 |
| | JEG3 | miR-218-1-3p mimics | IL-6 | 22.77 | 22.46 | 22.36 |
| | HTR-8 | miR-218-1-3p mimics | IL-6 | 22.12 | 22.45 | 22.26 |
| | JEG3 | NC mimics | GAPDH | 19.66 | 19.43 | 18.94 |
| | HTR-8 | NC mimics | GAPDH | 17.89 | 18.8 | 18.75 |
| | JEG3 | miR-218-1-3p mimics | GAPDH | 19.2 | 19.9 | 19.44 |
| | HTR-8 | miR-218-1-3p mimics | GAPDH | 17.85 | 18.55 | 18.26 |
| Fig. 1F | JEG3 | NC mimics | IL-1β | 23.57 | 23.95 | 23.9 |
| | HTR-8 | NC mimics | IL-1β | 21.6 | 21.43 | 21.37 |
| | JEG3 | miR-218-1-3p mimics | IL-1β | 22.19 | 22 | 21.77 |
| | HTR-8 | miR-218-1-3p mimics | IL-1β | 20.35 | 20.08 | 19.96 |
| | JEG3 | NC mimics | GAPDH | 19.92 | 19.44 | 19.36 |
| | HTR-8 | NC mimics | GAPDH | 18.76 | 18.5 | 17.95 |
| | JEG3 | miR-218-1-3p mimics | GAPDH | 18.93 | 19.25 | 19.37 |
| | HTR-8 | miR-218-1-3p mimics | GAPDH | 17.84 | 18.83 | 17.89 |
| Fig. 1G | JEG3 | NC mimics | TNF-α | 24.28 | 24.12 | 24.39 |
| | HTR-8 | NC mimics | TNF-α | 22.12 | 22.53 | 22.12 |
| | JEG3 | miR-218-1-3p mimics | TNF-α | 22.15 | 22.28 | 21.96 |
| | HTR-8 | miR-218-1-3p mimics | TNF-α | 21.49 | 21.07 | 21.26 |
| | JEG3 | NC mimics | GAPDH | 19.86 | 19.37 | 19.26 |
| | HTR-8 | NC mimics | GAPDH | 18.65 | 18.31 | 18.46 |
| | JEG3 | miR-218-1-3p mimics | GAPDH | 19.39 | 18.95 | 19.29 |
| | HTR-8 | miR-218-1-3p mimics | GAPDH | 18.73 | 18.76 | 18.55 |
| Fig. 1H | JEG3 | NC mimics | CXCL-8 | 24.86 | 25.02 | 24.81 |
| | HTR-8 | NC mimics | CXCL-8 | 23.84 | 23.65 | 23.77 |
| | JEG3 | miR-218-1-3p mimics | CXCL-8 | 23.44 | 23.79 | 23.63 |
| | HTR-8 | miR-218-1-3p mimics | CXCL-8 | 22.21 | 22.29 | 22.56 |
| | JEG3 | NC mimics | GAPDH | 19.16 | 19.06 | 19.29 |
| | HTR-8 | NC mimics | GAPDH | 18.12 | 18.69 | 18.43 |
| | JEG3 | miR-218-1-3p mimics | GAPDH | 19.59 | 19.69 | 19.35 |
| | HTR-8 | miR-218-1-3p mimics | GAPDH | 18.61 | 18.15 | 18.7 |
| Fig. 2F | JEG3 | pcDNA3.1 | miR-218-1-3p | 26.81 | 26.83 | 27.08 |
| | HTR-8 | pcDNA3.1 | miR-218-1-3p | 26.12 | 26.28 | 26.1 |
| | JEG3 | pcDNA3.1-JUN | miR-218-1-3p | 24.75 | 24.78 | 25.11 |
| | HTR-8 | pcDNA3.1-JUN | miR-218-1-3p | 24.52 | 24.48 | 24.8 |
| | JEG3 | pcDNA3.1 | U6 | 19.4 | 19.38 | 19.55 |
| | HTR-8 | pcDNA3.1 | U6 | 17.95 | 17.65 | 18.45 |
| | JEG3 | pcDNA3.1-JUN | U6 | 19.52 | 19.34 | 19.02 |
| | HTR-8 | pcDNA3.1-JUN | U6 | 17.56 | 18.28 | 18.36 |
| Fig. 2I | JEG3 | Control | miR-218-1-3p | 26.87 | 26.89 | 27.1 |
| | HTR-8 | Control | miR-218-1-3p | 27.17 | 26.83 | 26.66 |
| | JEG3 | TCS | miR-218-1-3p | 25.04 | 25.15 | 24.75 |
| | HTR-8 | TCS | miR-218-1-3p | 25.14 | 24.72 | 24.87 |
| | JEG3 | TCS+sh-JUN | miR-218-1-3p | 26.87 | 26.66 | 26.98 |
| | HTR-8 | TCS+sh-JUN | miR-218-1-3p | 26.71 | 26.54 | 26.31 |
| | JEG3 | Control | U6 | 19.09 | 18.78 | 19.4 |
| | HTR-8 | Control | U6 | 17.68 | 18.42 | 18.23 |
| | JEG3 | TCS | U6 | 19.5 | 18.97 | 19.46 |
| | HTR-8 | TCS | U6 | 18.12 | 18.07 | 18.05 |
| | JEG3 | TCS+sh-JUN | U6 | 18.9 | 19.61 | 18.7 |
| | HTR-8 | TCS+sh-JUN | U6 | 17.83 | 17.86 | 17.6 |
| Figure 3A | JEG3 | Control | PSME3 | 24.44 | 24.49 | 24.6 |
| | JEG3 | TCS | PSME3 | 25.03 | 25.3 | 25.12 |
| | JEG3 | Control | HOXA11 | 24.47 | 24.3 | 24.09 |
| | JEG3 | TCS | HOXA11 | 23.65 | 23.95 | 23.85 |
| | JEG3 | Control | CD8B | 22.92 | 22.91 | 22.69 |
| | JEG3 | TCS | CD8B | 23.53 | 23.18 | 23.42 |
| | JEG3 | Control | HK1 | 23.76 | 23.74 | 24.01 |
| | JEG3 | TCS | HK1 | 23.98 | 24.2 | 24.38 |

| | | | | | | |
|---|---|---|---|---|---|---|
| Figure 3A | JEG3 | Control | GALP | 22.47 | 22.57 | 22.38 |
| | JEG3 | TCS | GALP | 22.58 | 22.87 | 22.8 |
| | JEG3 | Control | HIST1H2AI | 24.55 | 24.65 | 24.76 |
| | JEG3 | TCS | HIST1H2AI | 24.71 | 25.14 | 25.03 |
| | JEG3 | Control | SLC35C1 | 21.29 | 21.43 | 21.25 |
| | JEG3 | TCS | SLC35C1 | 23.33 | 23.66 | 24.13 |
| | JEG3 | Control | GAPDH | 19.57 | 19.17 | 19.39 |
| | JEG3 | TCS | GAPDH | 19.88 | 19.54 | 19.64 |
| Figure 3B | HTR-8 | Control | CD8B | 25.11 | 25.1 | 24.82 |
| | HTR-8 | TCS | CD8B | 25.09 | 25.38 | 25.14 |
| | HTR-8 | Control | SLC35C1 | 24.07 | 23.79 | 24.13 |
| | HTR-8 | TCS | SLC35C1 | 25.35 | 25.72 | 25.62 |
| | HTR-8 | Control | GAPDH | 18.57 | 17.96 | 18.48 |
| | HTR-8 | TCS | GAPDH | 18.06 | 18.23 | 18.56 |
| Figure 3D | JEG3 | NC mimics | SLC35C1 | 21.93 | 22.08 | 22.13 |
| | HTR-8 | NC mimics | SLC35C1 | 23.51 | 23.63 | 23.66 |
| | JEG3 | miR-218-1-3p mimics | SLC35C1 | 24.03 | 24.19 | 24.29 |
| | HTR-8 | miR-218-1-3p mimics | SLC35C1 | 25.22 | 25.16 | 25.46 |
| | JEG3 | NC mimics | GAPDH | 19.37 | 19.68 | 19.43 |
| | HTR-8 | NC mimics | GAPDH | 17.84 | 18.73 | 18.55 |
| | JEG3 | miR-218-1-3p mimics | GAPDH | 19.13 | 19.18 | 19.61 |
| | HTR-8 | miR-218-1-3p mimics | GAPDH | 18.45 | 17.9 | 17.93 |
| Figure 3E (原 Figure 3F) | JEG3 | NC inhibitor | SLC35C1 | 21.19 | 21.38 | 21.13 |
| | HTR-8 | NC inhibitor | SLC35C1 | 23.93 | 23.94 | 24.11 |
| | JEG3 | miR-218-1-3p inhibitor | SLC35C1 | 18.13 | 17.89 | 18.22 |
| | HTR-8 | miR-218-1-3p inhibitor | SLC35C1 | 22.11 | 22.03 | 22.38 |
| | JEG3 | NC inhibitor | GAPDH | 19.66 | 19.57 | 19.87 |
| | HTR-8 | NC inhibitor | GAPDH | 17.95 | 18.59 | 18.41 |
| | JEG3 | miR-218-1-3p inhibitor | GAPDH | 19.07 | 18.94 | 19.35 |
| | HTR-8 | miR-218-1-3p inhibitor | GAPDH | 18.25 | 17.85 | 18.76 |
| Figure 4A | JEG3 | sh-NC | SLC35C1 | 22.01 | 21.61 | 21.94 |
| | HTR-8 | sh-NC | SLC35C1 | 23.77 | 24.28 | 24.1 |
| | JEG3 | sh-SLC35C1#1 | SLC35C1 | 24.35 | 24.08 | 23.94 |
| | HTR-8 | sh-SLC35C1#1 | SLC35C1 | 25.83 | 25.48 | 25.8 |
| | JEG3 | sh-SLC35C1#2 | SLC35C1 | 23.4 | 23.03 | 23.28 |
| | HTR-8 | sh-SLC35C1#2 | SLC35C1 | 25.48 | 25.19 | 25.43 |
| | JEG3 | sh-NC | GAPDH | 19.51 | 19.77 | 19.44 |
| | HTR-8 | sh-NC | GAPDH | 18.14 | 18.09 | 18.48 |
| | JEG3 | sh-SLC35C1#1 | GAPDH | 19.14 | 19.59 | 19.75 |
| | HTR-8 | sh-SLC35C1#1 | GAPDH | 18.31 | 18.3 | 17.88 |
| | JEG3 | sh-SLC35C1#2 | GAPDH | 19.52 | 19.64 | 19.07 |
| | HTR-8 | sh-SLC35C1#2 | GAPDH | 18.13 | 18.7 | 18.39 |
| Fig. 4D | JEG3 | sh-NC | IL-6 | 23.18 | 23.16 | 23.33 |
| | HTR-8 | sh-NC | IL-6 | 22.53 | 22.7 | 22.78 |
| | JEG3 | sh-SLC35C1#1 | IL-6 | 22.56 | 22.29 | 22.59 |
| | HTR-8 | sh-SLC35C1#1 | IL-6 | 21.94 | 21.73 | 21.53 |
| | JEG3 | sh-NC | GAPDH | 19.74 | 18.98 | 19.44 |
| | HTR-8 | sh-NC | GAPDH | 18.78 | 18.42 | 17.97 |
| | JEG3 | sh-SLC35C1#1 | GAPDH | 19.39 | 19.88 | 19.68 |
| | HTR-8 | sh-SLC35C1#1 | GAPDH | 18.45 | 18.79 | 18.04 |
| | JEG3 | sh-NC | IL-1β | 24.03 | 24.48 | 24.34 |
| | HTR-8 | sh-NC | IL-1β | 22.27 | 21.93 | 21.91 |
| | JEG3 | sh-SLC35C1#1 | IL-1β | 23.35 | 23.12 | 23.38 |
| | HTR-8 | sh-SLC35C1#1 | IL-1β | 20.69 | 20.94 | 20.54 |
| | JEG3 | sh-NC | GAPDH | 19.8 | 19.63 | 19.5 |
| | HTR-8 | sh-NC | GAPDH | 18.25 | 18.15 | 17.97 |
| | JEG3 | sh-SLC35C1#1 | GAPDH | 19.73 | 19.75 | 19.45 |
| | HTR-8 | sh-SLC35C1#1 | GAPDH | 18.28 | 18.34 | 17.96 |
| | JEG3 | sh-NC | TNF-α | 23.6 | 23.79 | 23.93 |
| | HTR-8 | sh-NC | TNF-α | 22.7 | 22.52 | 22.82 |
| | JEG3 | sh-SLC35C1#1 | TNF-α | 21.76 | 21.98 | 22.11 |
| | HTR-8 | sh-SLC35C1#1 | TNF-α | 20.29 | 20.61 | 20.39 |
| | JEG3 | sh-NC | GAPDH | 19.69 | 19.08 | 19.4 |
| | HTR-8 | sh-NC | GAPDH | 18.37 | 18.04 | 18.37 |
| | JEG3 | sh-SLC35C1#1 | GAPDH | 19.68 | 19.57 | 19.84 |
| | HTR-8 | sh-SLC35C1#1 | GAPDH | 18.13 | 17.93 | 18.33 |
| | JEG3 | sh-NC | CXCL-8 | 24.36 | 24.38 | 24.09 |
| | HTR-8 | sh-NC | CXCL-8 | 22.88 | 23.01 | 23.04 |
| | JEG3 | sh-SLC35C1#1 | CXCL-8 | 22.45 | 22.71 | 22.8 |
| | HTR-8 | sh-SLC35C1#1 | CXCL-8 | 20.93 | 21.25 | 21.13 |
| | JEG3 | sh-NC | GAPDH | 19.34 | 19.3 | 19.87 |
| | HTR-8 | sh-NC | GAPDH | 18.01 | 18.19 | 18.48 |
| | JEG3 | sh-SLC35C1#1 | GAPDH | 18.97 | 19.8 | 19.27 |
| | HTR-8 | sh-SLC35C1#1 | GAPDH | 17.89 | 18.48 | 18.07 |
| | JEG3 | Control | SLC35C1 | 22.17 | 22.04 | 21.98 |
| | HTR-8 | Control | SLC35C1 | 23.83 | 24.06 | 24 |
| | JEG3 | TCS | SLC35C1 | 24.05 | 24.38 | 24.44 |
| | HTR-8 | TCS | SLC35C1 | 25.62 | 25.93 | 25.96 |
| | JEG3 | TCS+miR-218-1-3p inhibitor | SLC35C1 | 22.57 | 22.5 | 22.79 |

| | | | | | | |
|---|---|---|---|---|---|---|
| Fig. 5A | HTR-8 | TCS+miR-218-1-3p inhibitor | SLC35C1 | 24.05 | 24.36 | 24.25 |
| | JEG3 | TCS+miR-218-1-3p inhibitor+sh-SLC35C1#1 | SLC35C1 | 24.18 | 24.08 | 23.8 |
| | HTR-8 | TCS+miR-218-1-3p inhibitor+sh-SLC35C1#1 | SLC35C1 | 25.8 | 25.51 | 25.42 |
| | JEG3 | Control | GAPDH | 19.24 | 19.05 | 19.49 |
| | HTR-8 | Control | GAPDH | 18.66 | 18.66 | 17.85 |
| | JEG3 | TCS | GAPDH | 19.42 | 19 | 19.31 |
| | HTR-8 | TCS | GAPDH | 18.13 | 18.51 | 18.47 |
| | JEG3 | TCS+miR-218-1-3p inhibitor | GAPDH | 19.85 | 19.84 | 19.42 |
| | HTR-8 | TCS+miR-218-1-3p inhibitor | GAPDH | 18.64 | 18.77 | 18.4 |
| | JEG3 | TCS+miR-218-1-3p inhibitor+sh-SLC35C1#1 | GAPDH | 19.64 | 19.5 | 19.51 |
| | HTR-8 | TCS+miR-218-1-3p inhibitor+sh-SLC35C1#1 | GAPDH | 18.67 | 17.93 | 18.14 |
| Fig. 5D | JEG3 | Control | IL-6 | 23.84 | 23.63 | 23.77 |
| | HTR-8 | Control | IL-6 | 22.9 | 22.37 | 22.66 |
| | JEG3 | TCS | IL-6 | 22.39 | 22.14 | 22.07 |
| | HTR-8 | TCS | IL-6 | 22 | 22.37 | 22.18 |
| | JEG3 | TCS+miR-218-1-3p inhibitor | IL-6 | 23.28 | 23.53 | 23.64 |
| | HTR-8 | TCS+miR-218-1-3p inhibitor | IL-6 | 22.77 | 22.97 | 23.08 |
| | JEG3 | TCS+miR-218-1-3p inhibitor+sh-SLC35C1#1 | IL-6 | 21.93 | 22.15 | 22.25 |
| | HTR-8 | TCS+miR-218-1-3p inhibitor+sh-SLC35C1#1 | IL-6 | 21.76 | 22.05 | 21.87 |
| | JEG3 | Control | GAPDH | 19.89 | 19.64 | 19.49 |
| | HTR-8 | Control | GAPDH | 18.06 | 18.09 | 18.27 |
| | JEG3 | TCS | GAPDH | 19.2 | 19.85 | 19.3 |
| | HTR-8 | TCS | GAPDH | 18.55 | 18.47 | 18.45 |
| | JEG3 | TCS+miR-218-1-3p inhibitor | GAPDH | 19.87 | 19.03 | 19.33 |
| | HTR-8 | TCS+miR-218-1-3p inhibitor | GAPDH | 17.9 | 18.65 | 18.64 |
| | JEG3 | TCS+miR-218-1-3p inhibitor+sh-SLC35C1#1 | GAPDH | 19.28 | 19.74 | 19.15 |
| | HTR-8 | TCS+miR-218-1-3p inhibitor+sh-SLC35C1#1 | GAPDH | 18.59 | 18.06 | 17.99 |
| Fig. 5E | JEG3 | Control | IL-1β | 23.71 | 23.9 | 23.91 |
| | HTR-8 | Control | IL-1β | 22.37 | 22 | 22.03 |
| | JEG3 | TCS | IL-1β | 22.78 | 23.08 | 22.84 |
| | HTR-8 | TCS | IL-1β | 21.16 | 20.96 | 21.35 |
| | JEG3 | TCS+miR-218-1-3p inhibitor | IL-1β | 23.58 | 23.74 | 23.4 |
| | HTR-8 | TCS+miR-218-1-3p inhibitor | IL-1β | 22.13 | 21.99 | 21.74 |
| | JEG3 | TCS+miR-218-1-3p inhibitor+sh-SLC35C1#1 | IL-1β | 23.01 | 22.7 | 22.66 |
| | HTR-8 | TCS+miR-218-1-3p inhibitor+sh-SLC35C1#1 | IL-1β | 21.26 | 21.56 | 21.34 |
| | JEG3 | Control | GAPDH | 19.45 | 19.2 | 19.39 |
| | HTR-8 | Control | GAPDH | 18.63 | 18.03 | 18.24 |
| | JEG3 | TCS | GAPDH | 19.31 | 19.28 | 19.5 |
| | HTR-8 | TCS | GAPDH | 18.08 | 18.48 | 18.02 |
| | JEG3 | TCS+miR-218-1-3p inhibitor | GAPDH | 19.48 | 19.27 | 18.97 |
| | HTR-8 | TCS+miR-218-1-3p inhibitor | GAPDH | 18 | 18.36 | 18.3 |
| | JEG3 | TCS+miR-218-1-3p inhibitor+sh-SLC35C1#1 | GAPDH | 19.45 | 19.35 | 18.95 |
| | HTR-8 | TCS+miR-218-1-3p inhibitor+sh-SLC35C1#1 | GAPDH | 17.92 | 18.79 | 18.59 |
| Fig. 5F | JEG3 | Control | TNF-α | 24.64 | 24.39 | 24.16 |
| | HTR-8 | Control | TNF-α | 22.65 | 22.78 | 22.99 |
| | JEG3 | TCS | TNF-α | 23.29 | 22.91 | 23.12 |
| | HTR-8 | TCS | TNF-α | 20.76 | 21.09 | 20.87 |
| | JEG3 | TCS+miR-218-1-3p inhibitor | TNF-α | 24.78 | 24.52 | 24.83 |
| | HTR-8 | TCS+miR-218-1-3p inhibitor | TNF-α | 22.6 | 22.99 | 22.65 |
| | JEG3 | TCS+miR-218-1-3p inhibitor+sh-SLC35C1#1 | TNF-α | 22.81 | 22.42 | 22.67 |
| | HTR-8 | TCS+miR-218-1-3p inhibitor+sh-SLC35C1#1 | TNF-α | 21.14 | 21.41 | 21 |
| | JEG3 | Control | GAPDH | 19.33 | 19.14 | 18.95 |
| | HTR-8 | Control | GAPDH | 18.07 | 18.64 | 18.27 |
| | JEG3 | TCS | GAPDH | 19.61 | 19.42 | 19.82 |
| | HTR-8 | TCS | GAPDH | 18.26 | 18.8 | 18.22 |
| | JEG3 | TCS+miR-218-1-3p inhibitor | GAPDH | 19.83 | 19.39 | 19.46 |
| | HTR-8 | TCS+miR-218-1-3p inhibitor | GAPDH | 18.51 | 18.54 | 17.89 |
| | JEG3 | TCS+miR-218-1-3p inhibitor+sh-SLC35C1#1 | GAPDH | 19.08 | 19.76 | 18.97 |
| | HTR-8 | TCS+miR-218-1-3p inhibitor+sh-SLC35C1#1 | GAPDH | 18.79 | 18.55 | 18.22 |
| | JEG3 | Control | CXCL-8 | 24.29 | 24.1 | 24.12 |
| | HTR-8 | Control | CXCL-8 | 23.18 | 23.2 | 22.99 |
| | JEG3 | TCS | CXCL-8 | 22.65 | 22.27 | 22.4 |
| | HTR-8 | TCS | CXCL-8 | 21 | 20.98 | 21.37 |

| | | | | | | |
|---|---|---|---|---|---|---|
| Fig. 5G | JEG3 | TCS+miR-218-1-3p inhibitor | CXCL-8 | 23.95 | 24.3 | 24.03 |
| | HTR-8 | TCS+miR-218-1-3p inhibitor | CXCL-8 | 22.57 | 22.84 | 22.67 |
| | JEG3 | TCS+miR-218-1-3p inhibitor+sh-SLC35C1#1 | CXCL-8 | 22.68 | 22.94 | 22.52 |
| | HTR-8 | TCS+miR-218-1-3p inhibitor+sh-SLC35C1#1 | CXCL-8 | 20.99 | 21.22 | 21.24 |
| | JEG3 | Control | GAPDH | 19.81 | 19.38 | 19.53 |
| | HTR-8 | Control | GAPDH | 18.78 | 18.52 | 18.43 |
| | JEG3 | TCS | GAPDH | 19.16 | 19.1 | 19.24 |
| | HTR-8 | TCS | GAPDH | 17.85 | 18.48 | 18.81 |
| | JEG3 | TCS+miR-218-1-3p inhibitor | GAPDH | 19.87 | 19.7 | 19.1 |
| | HTR-8 | TCS+miR-218-1-3p inhibitor | GAPDH | 18.75 | 18.28 | 18.26 |
| | JEG3 | TCS+miR-218-1-3p inhibitor+sh-SLC35C1#1 | GAPDH | 19.16 | 19.52 | 19.8 |
| | HTR-8 | TCS+miR-218-1-3p inhibitor+sh-SLC35C1#1 | GAPDH | 18.01 | 18.4 | 18.31 |
| Figure S1D | JEG3 | Control | IL-6 | 24.22 | 23.86 | 23.67 |
| | HTR-8 | Control | IL-6 | 22.99 | 22.85 | 22.81 |
| | JEG3 | TCS | IL-6 | 23.44 | 23.25 | 23.09 |
| | HTR-8 | TCS | IL-6 | 22.93 | 22.67 | 22.53 |
| | JEG3 | Control | GAPDH | 19.11 | 19.26 | 19.67 |
| | HTR-8 | TCS | GAPDH | 18.55 | 18.48 | 18.43 |
| Figure S1E | JEG3 | Control | IL-1β | 23.93 | 23.95 | 23.72 |
| | HTR-8 | Control | IL-1β | 22.03 | 21.81 | 22.15 |
| | JEG3 | TCS | IL-1β | 22.79 | 22.92 | 23.15 |
| | HTR-8 | TCS | IL-1β | 21.07 | 20.76 | 21.11 |
| | JEG3 | Control | GAPDH | 19.4 | 19.35 | 19.17 |
| | HTR-8 | TCS | GAPDH | 18.53 | 18.4 | 18 |
| Figure S1F | JEG3 | Control | TNF-α | 24.2 | 23.95 | 24.26 |
| | HTR-8 | Control | TNF-α | 23.03 | 23.25 | 22.97 |
| | JEG3 | TCS | TNF-α | 22.38 | 22.6 | 22.21 |
| | HTR-8 | TCS | TNF-α | 21.96 | 22.29 | 21.99 |
| | JEG3 | Control | GAPDH | 18.95 | 19.14 | 19.85 |
| | HTR-8 | TCS | GAPDH | 18.19 | 18.78 | 18.83 |
| Figure S1G | JEG3 | Control | CXCL-8 | 24.9 | 24.8 | 25.09 |
| | HTR-8 | Control | CXCL-8 | 23.84 | 23.77 | 23.63 |
| | JEG3 | TCS | CXCL-8 | 23.25 | 22.87 | 22.9 |
| | HTR-8 | TCS | CXCL-8 | 22.62 | 22.71 | 22.36 |
| | JEG3 | Control | GAPDH | 18.95 | 19.85 | 19.37 |
| | HTR-8 | TCS | GAPDH | 18.34 | 18.02 | 18.24 |
